# Design and Validation of a PU–Six-Cavity Helmholtz Metamaterial Composite Acoustic Package for Broadband Noise Reduction in Commercial Vehicle Cabs

**DOI:** 10.3390/ma19163490

**Published:** 2026-08-18

**Authors:** Chi Cai, Yasi Duan, Tianjin Wang, An Wang, Xiao Wang, Yuanyuan Shi, Xikang Xiao, Yizhe Huang

**Affiliations:** 1 Hubei Key Laboratory of Modern Manufacturing Quality Engineering, School of Mechanical Engineering, Hubei University of Technology, Wuhan 430068, China; 102500042@hbut.edu.cn (C.C.); 2110100426@hbut.edu.cn (T.W.); 102500010@hbut.edu.cn (A.W.); 102310162@hbut.edu.cn (X.W.); 2 School of Intelligent Manufacturing, Hubei University, Wuhan 430062, China; 202531129012099@stu.hubu.edu.cn (Y.D.); yyshi@hubu.edu.cn (Y.S.); 3 Dongfeng Liuzhou Motor Co., Ltd., Liuzhou 545005, China

**Keywords:** commercial vehicle cab, acoustic metamaterial, broadband noise reduction, Helmholtz resonator, PU porous layer, composite acoustic package

## Abstract

To address the broadband noise distribution, complex excitation sources, and insufficient low-frequency attenuation of conventional porous acoustic packages in commercial vehicle cabs, this study proposes a PU–six-cavity Helmholtz metamaterial composite acoustic package for broadband noise reduction. The proposed structure consists of a 30 mm PU porous layer for mid-to-high-frequency dissipation and a 30 mm six-cavity Helmholtz metamaterial layer for low-frequency absorption, forming a 60 mm composite acoustic package. A full-vehicle acoustic model of a commercial vehicle cab was established in VA One to identify the A-weighted sound pressure level (SPL) spectrum at the driver position. The results show that the PU porous acoustic package improves the mid- and high-frequency noise response, whereas pronounced peaks remain in the low- and low-to-mid-frequency ranges. To enhance these bands, the six sub-cavities of the Helmholtz metamaterial were tuned to 200, 250, 315, 400, 500, and 630 Hz through spatial partitioning and cavity grouping. COMSOL (version 6.1) simulations and particle velocity distributions confirmed the multi-peak absorption mechanism and the selective excitation of the corresponding sub-cavities. The composite structure was further validated through impedance-tube measurements, full-vehicle acoustic simulations, and in-vehicle tests. The VA One simulation shows that the total A-weighted SPL at the driver position decreases from 68.25 dB for the PU porous package to 66.01 dB after introducing the six-cavity Helmholtz metamaterial, corresponding to an additional reduction of 2.24 dB. A preliminary in-vehicle test under a stationary idling condition shows that the total A-weighted SPL near the driver’s ear decreases from 55.83 dB(A) to 54.56 dB(A). These results demonstrate that the proposed PU–six-cavity Helmholtz metamaterial composite acoustic package combines broadband porous dissipation with low-frequency resonant absorption, providing a feasible solution for broadband noise control in commercial vehicle cabs.

## 1. Introduction

With the wide application of commercial vehicles in logistics, construction, and public-service scenarios, the acoustic environment inside their cabs has become an important indicator for evaluating overall vehicle NVH (Noise, Vibration and Harshness) performance and ride comfort [1]. Compared with passenger vehicles, commercial vehicles generally use high-displacement diesel engines and operate under heavier loads, producing stronger structural vibration and aerodynamic noise during service. In addition, the cab has a larger structural size and a greater number of panels, while noise sources such as the powertrain, intake and exhaust systems, and tire–road interaction act simultaneously. As a result, the interior noise of commercial vehicle cabs exhibits multi-source excitation, broadband distribution, and complex coupled propagation. Long-term exposure to high noise levels not only reduces driver comfort but may also adversely affect hearing health, concentration, and driving safety. Therefore, effective noise control under constraints of limited installation space and vehicle mass remains a key issue in commercial vehicle NVH design [2].

Passive acoustic packages are among the most widely used technologies for vehicle interior noise control [3]. Typical sound-absorbing materials include porous materials such as polyurethane (PU) foam and melamine foam (MF). These materials are lightweight, easy to process, flexible in installation, and generally effective in the mid- and high-frequency ranges; hence, they are widely used in automotive interior trims and sound-insulation structures [4,5]. However, the absorption performance of a single porous material is strongly frequency dependent. In particular, low-frequency absorption is restricted by material thickness, pore scale, and acoustic wavelength, resulting in insufficient attenuation in the low-frequency range [6,7,8]. Therefore, for low-frequency noise reduction in commercial vehicle cabs, it is necessary to introduce functional structures with low-frequency modulation capability on the basis of a conventional PU porous absorbing layer.

In recent years, acoustic metamaterials based on local resonance mechanisms have provided new structural design strategies for low-frequency noise control [9,10,11]. Among them, Helmholtz resonant structures generate resonant absorption through the coupled vibration between the air mass in the neck and the air compliance in the cavity, enabling efficient acoustic-energy dissipation near specific frequencies [12,13]. By properly adjusting the neck cross-sectional area, effective neck length, and backing-cavity volume, low-frequency absorption can be achieved within a relatively compact structural size. Therefore, Helmholtz-type acoustic metamaterials have promising application potential in vehicle NVH, duct noise control, and acoustic package design under limited installation space [14,15].

From the theoretical perspective, Morse and Ingard [12] systematically described the mechanism of Helmholtz resonance in classical acoustic theory and showed that a Helmholtz resonator can be represented as an equivalent mass–spring–damper system consisting of the acoustic mass in the neck, the acoustic compliance of the cavity, and thermoviscous losses. Selamet and Lee [13] further clarified the extended-neck effect of Helmholtz resonators, which is important for predicting the effective neck length and resonance shift. With the development of artificial periodic structures and subwavelength acoustic units, the concept of acoustic metamaterials has gradually emerged and rapidly expanded. Liu et al. [9] proposed locally resonant sonic materials and demonstrated that artificial subwavelength structures can overcome the scale limitation of conventional acoustic materials, enabling effective manipulation of low-frequency sound waves. Fang et al. [10] further developed acoustic metamaterials with negative bulk moduli using Helmholtz resonator arrays, thereby establishing an important foundation for low-frequency acoustic control based on resonant units. Subsequent studies on coupled-resonator metasurfaces further broadened the design space for subwavelength sound absorption [11].

With further development, research interest has gradually shifted from single local resonant units to broadband, multi-peak, and engineering-oriented composite structures. Chang et al. [14] summarized intelligent materials and acoustic metamaterials for low-frequency sound absorption, including Helmholtz resonators, micro-perforated panels, porous media, membrane-type structures, and tunable structures. Recent reviews have also emphasized that combining porous dissipation, local resonance, and manufacturable structural design is an important route toward practical broadband low-frequency absorbers [15]. Ji et al. [16] and Long et al. [17] demonstrated that coupled metasurface units and bright–dark-mode coupling can broaden low-frequency absorption bands. Shen et al. [18] proposed a broadband low-frequency acoustic metamuffler, and Ma et al. [19] realized broadband low-frequency absorption using a two-port micro-perforated panel resonator. Duan et al. [20] proposed a low-frequency acoustic metamaterial based on the parallel connection of multiple spiral chambers, showing that multi-cavity coupling can effectively broaden the narrowband absorption range of single-cavity resonators. Ren et al. [21] developed a multifunctional metastructure for low-frequency broadband sound absorption and crash energy dissipation, while Liu et al. [22] improved absorption through energy dissipation in perforated porous materials backed by resonant cavities. More recently, Zhang and Xin [23] proposed Helmholtz resonators with porous material lining for broadband low-frequency sound absorption, Su et al. [24] developed a hybrid porous Helmholtz resonator optimized for low-frequency broadband absorption, Chen et al. [25] proposed compact Helmholtz resonators with side slits for low-frequency absorption, and Hedayati and Lakshmanan [26] reported an active Helmholtz-resonator-based acoustic metamaterial for adaptive broadband noise control. In addition, Choi and Jeon [27] achieved near-perfect absorption using hybrid resonance between subwavelength Helmholtz resonators with non-uniformly partitioned cavities, and recent studies on additive manufacturing, extended-neck resonators, and curled acoustic metasurfaces have further improved the compactness and fabrication feasibility of low-frequency absorbers [28,29,30].

Existing studies have shown that Helmholtz resonators and their arrays can realize low-frequency absorption at subwavelength scales [31]. Nevertheless, a single resonator usually exhibits a narrowband response, while multi-cavity structures are constrained by finite thickness, cavity-volume allocation, and manufacturing and sealing reliability [32]. For commercial vehicle cabs, where low-frequency peaks are scattered and installation space is limited, a low-frequency enhancement structure is still needed that can cover multiple target frequencies within a finite thickness and can be integrated with a conventional PU porous acoustic package.

In addition to geometric design, environmental and multiphysics effects should be considered for polymer-based or 3D-printed acoustic metamaterials used in vehicles. Temperature fluctuations may induce small geometric deformations or material-property variations, resulting in shifts in absorption peaks and deviations between theoretical, numerical, and experimental results [33]. Recent studies on 3D-printed multifunctional acoustic metamaterials have also shown that mechanical deformation resilience and structural stability are important for practical engineering applications [34]. Therefore, compactness, manufacturability, and service stability should be considered together when designing low-frequency metamaterial absorbers for vehicle cabs.

To address these challenges, this study proposes a PU–six-cavity Helmholtz metamaterial composite acoustic package. The composite package consists of a PU porous absorbing layer and a six-cavity inner–outer sector Helmholtz acoustic metamaterial. The PU porous layer mainly provides broadband dissipation in the mid- and high-frequency ranges, whereas the six-cavity Helmholtz metamaterial uses spatial partitioning into outer low-frequency cavities and inner mid-frequency cavities. The sub-cavities are tuned to 200, 250, 315, 400, 500, and 630 Hz, respectively, thereby generating multi-peak enhanced absorption in the low- and low-to-mid-frequency ranges. Compared with a single PU porous acoustic package, the proposed composite structure maintains mid-to-high-frequency absorption while improving energy dissipation at low-frequency peaks.

The novelty of this work lies not in using a Helmholtz resonator alone, but in integrating a limited-thickness six-cavity inner–outer sector resonant unit with a conventional PU porous acoustic package for commercial vehicle cab applications. Compared with previously reported single resonators, porous-lined resonators, or general multi-cavity absorbers, the proposed design establishes a complete material-to-vehicle validation route, including single-cavity tuning rules, six-cavity multi-peak design, impedance-tube verification, measured absorption-coefficient import into VA One, and in-vehicle noise reduction validation.

The main work of this paper is summarized as follows. First, a six-cavity inner–outer sector Helmholtz acoustic metamaterial is proposed, in which outer low-frequency cavities and inner mid-frequency cavities are integrated within a limited thickness to achieve structural tuning at multiple target frequencies. Second, a design procedure from embedded-neck single-cavity parametric simulation to six-cavity multi-peak structural design is established, and the effects of neck radius, cavity height, and neck depth on resonance frequency and absorption peak shape are clarified. Third, a composite acoustic package based on the concept of ‘PU porous absorption plus low-frequency enhancement by a six-cavity Helmholtz metamaterial’ is developed and validated through synergistic absorption simulations, impedance-tube measurements, full-vehicle acoustic simulations, and vehicle tests.

## 2. Noise Analysis of the Commercial Vehicle Cab with a Polyurethane Foam Porous Layer

### 2.1. Full-Vehicle Acoustic Model of the Commercial Vehicle Cab

**Figure 1:**
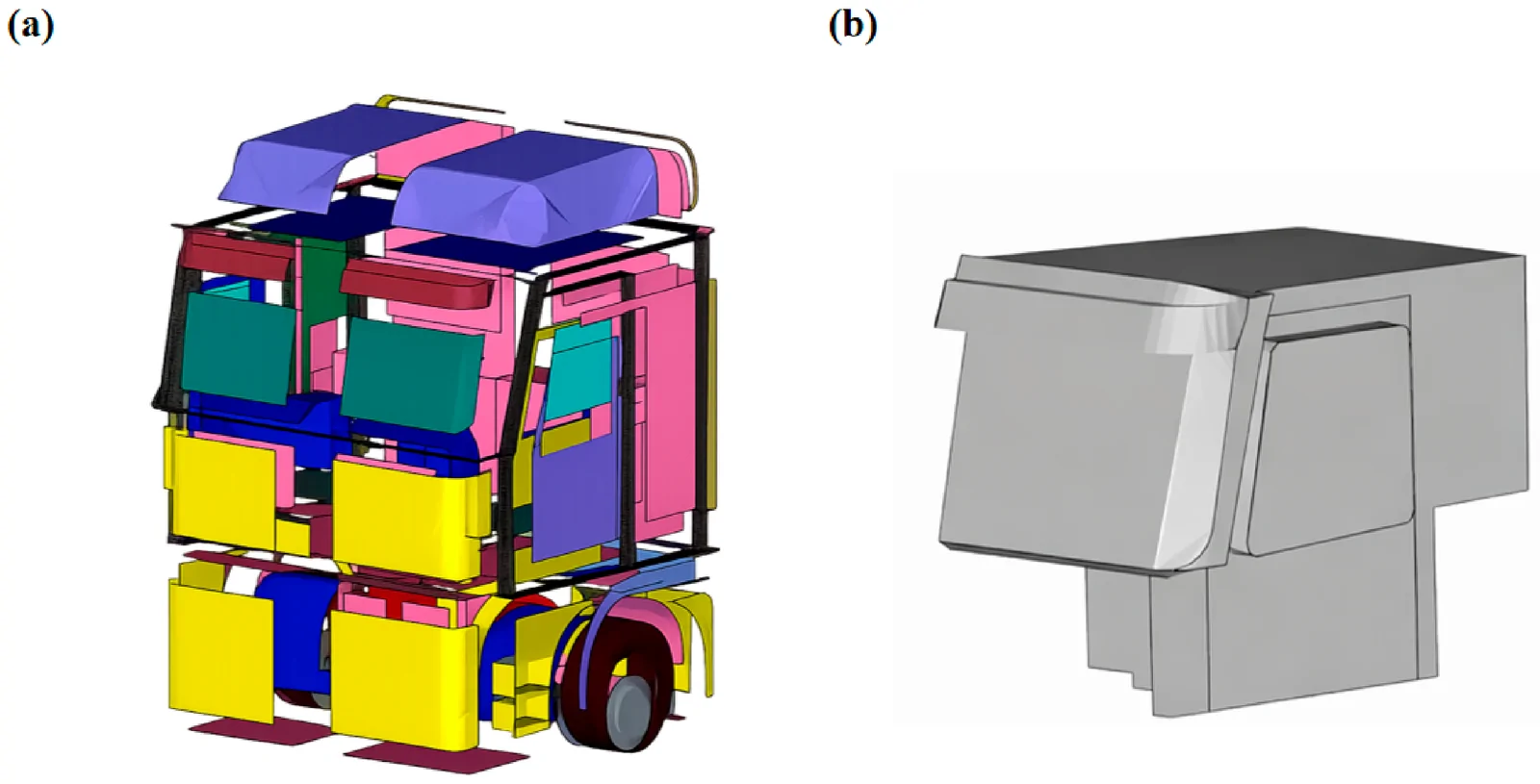
Acoustic modeling of the commercial vehicle cab: (**a**) full-vehicle FE–SEA model; (**b**) extracted driver’s acoustic cavity model.

To characterize the noise spectrum inside the commercial vehicle cab and to provide a unified baseline for evaluating the subsequent PU porous layer and composite acoustic package, a cab acoustic analysis model was established in VA One. The model was mainly used to extract the A-weighted SPL spectrum at the driver position and to compare different acoustic package schemes at the full-vehicle level. Considering that the cab noise covers low-, mid-, and high-frequency ranges, a hybrid FE–SEA method was adopted in the 200–1000 Hz range to describe the coupling response between the structure and the acoustic cavity, whereas SEA was used in the 1000–8000 Hz range to capture mid-to-high-frequency energy transmission. During model construction, the main vibro-acoustic transmission paths, including the front panel, floor, side panels, roof, and rear panel, were retained, while local accessories with negligible contributions to interior noise, such as windshield wipers and rearview mirrors, were simplified. This treatment balances model accuracy and computational efficiency. Figure 1 shows the full-vehicle acoustic model of the commercial vehicle cab and the position of the driver’s acoustic cavity.

### 2.2. Baseline Noise Response of the Body-in-White Cab

On the basis of the above model, the body-in-white (BIW) condition without any acoustic treatment was first used as the baseline, and the A-weighted SPL spectrum at the driver position was calculated. To be consistent with standard vehicle noise evaluation practice and the frequency-band statistical requirements of mid-frequency energy-based modeling, a 1/3-octave band division was adopted, and the analysis frequency range was set to 200–8000 Hz. This frequency range covers the dominant contribution bands of structure–cavity coupled noise in the cab, as well as the mid-to-low- and mid-to-high-frequency ranges in which the PU porous material and the subsequent composite acoustic package can be effective. It should be noted that noise below 200 Hz is usually more closely related to rigid-body vibration of the powertrain, mounting systems, and low-order body structural modes, which are more appropriately controlled by vibration isolation, structural optimization, or active control. Since this study focuses on a passive composite acoustic package with limited thickness, the low-frequency enhancement target is set above 200 Hz.

**Figure 2:**
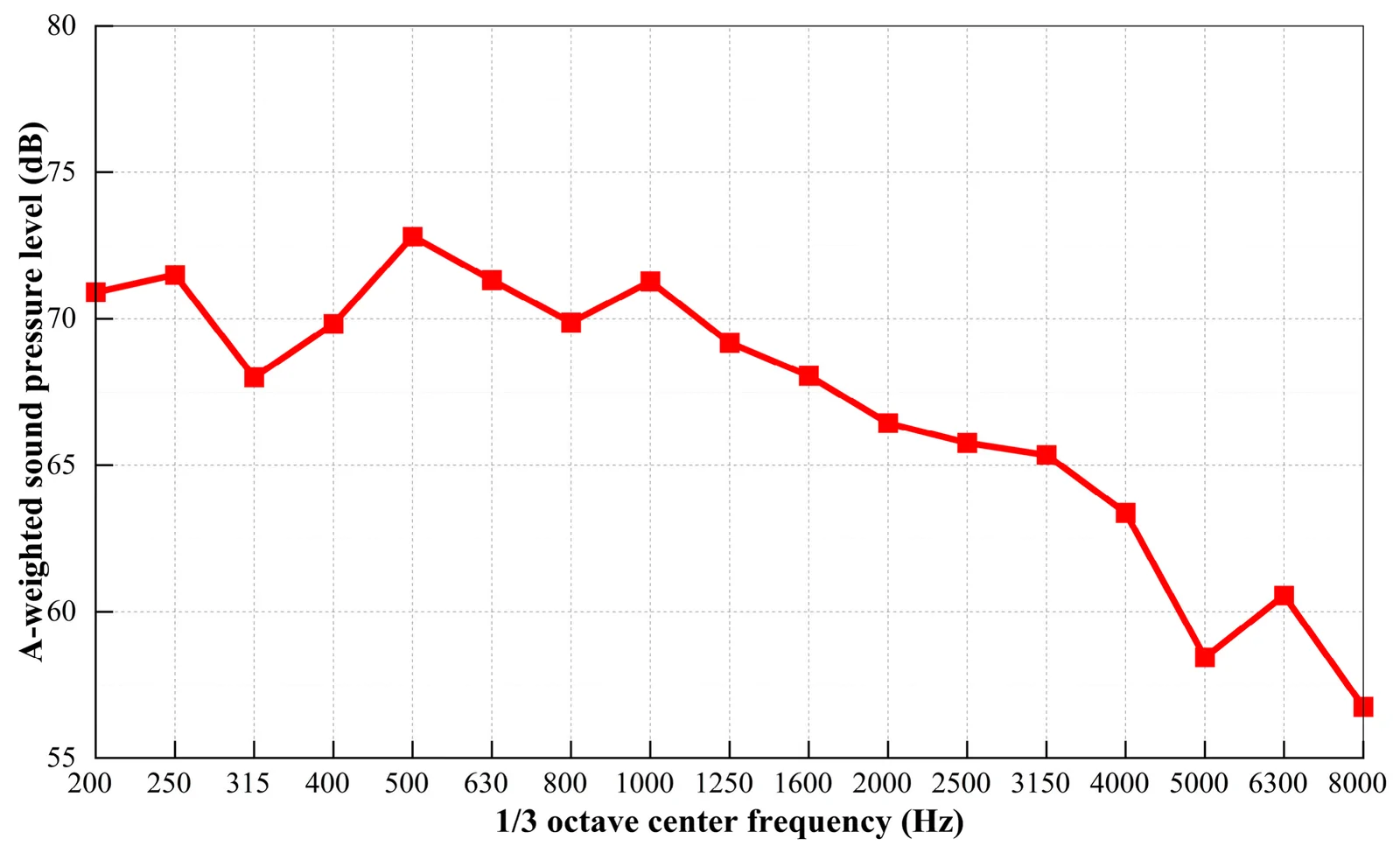
A-weighted SPL spectrum of the driver’s acoustic cavity under the BIW condition.

The A-weighted SPL spectrum of the driver’s acoustic cavity under the BIW condition is shown in Figure 2. Without any sound-absorbing material, the overall A-weighted SPL at the driver position is relatively high, with a total SPL of 81.1059 dB. Distinct peaks appear around 250, 500, and 1000 Hz, indicating that structural vibration transmission and structure–cavity coupling have important effects on the noise level at the driver position. Combined with the acoustic energy contribution analysis, the floor, front panel, side panels, and sleeper region are identified as key regions for acoustic package application. Accordingly, the PU porous layer and the PU–six-cavity Helmholtz composite acoustic package were preferentially applied to these high-contribution regions to improve material utilization and targeted noise reduction.

### 2.3. SPL Response with the PU Acoustic Package

Typical porous sound-absorbing materials include PU foam and MF. These materials have low density, favorable sound absorption performance, and good structural flexibility, and are therefore widely used in automotive interior trim and sound-insulation structures. Considering that PU foam can provide relatively effective acoustic-energy dissipation in the mid- and high-frequency ranges, PU foam was selected as the baseline acoustic package material in this study. The main acoustic parameters of the PU porous material used in this study are listed in Table 1.

**Figure 3:**
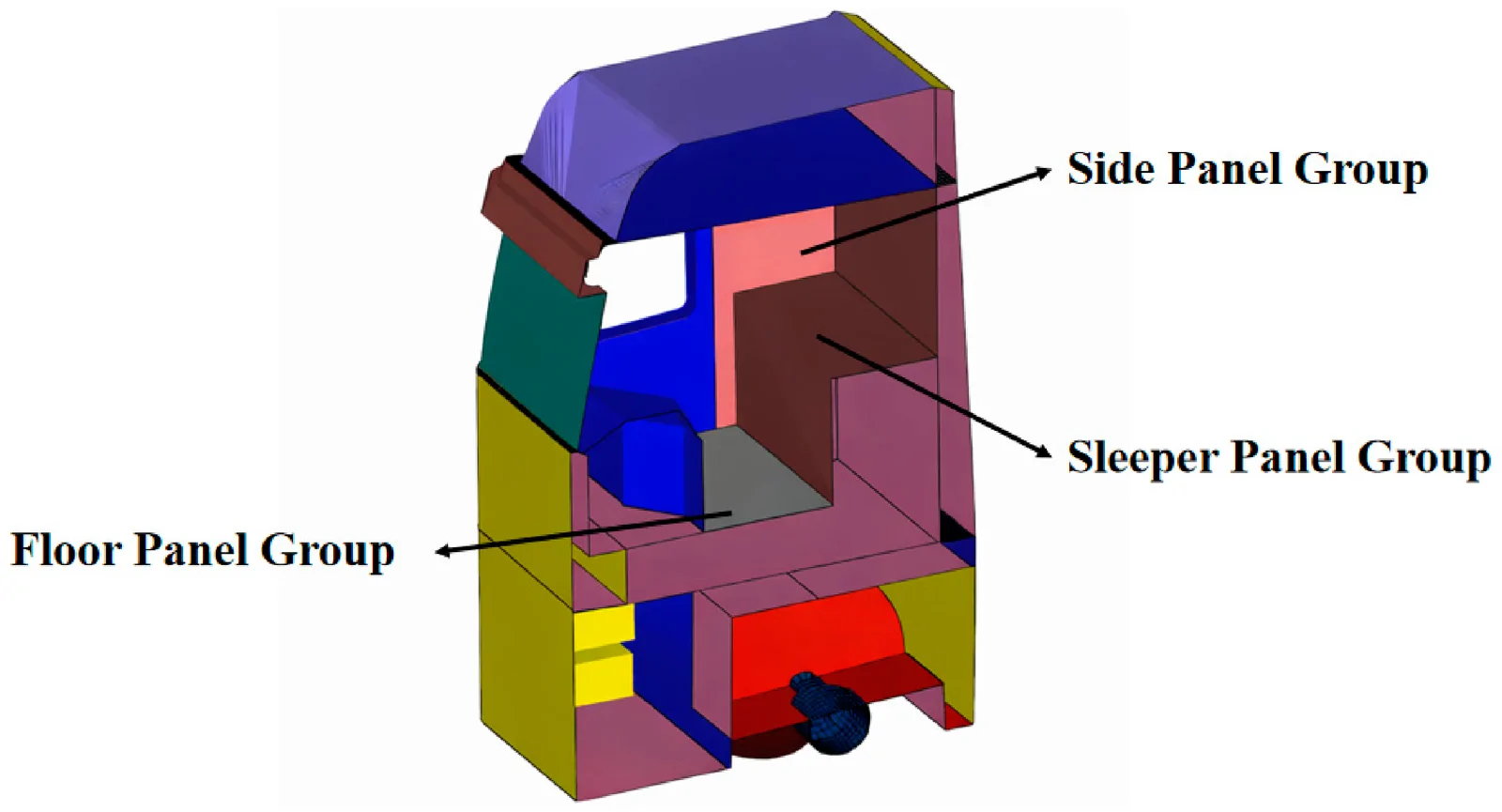
Coverage regions of the acoustic package on the cab panels.

**Figure 4:**
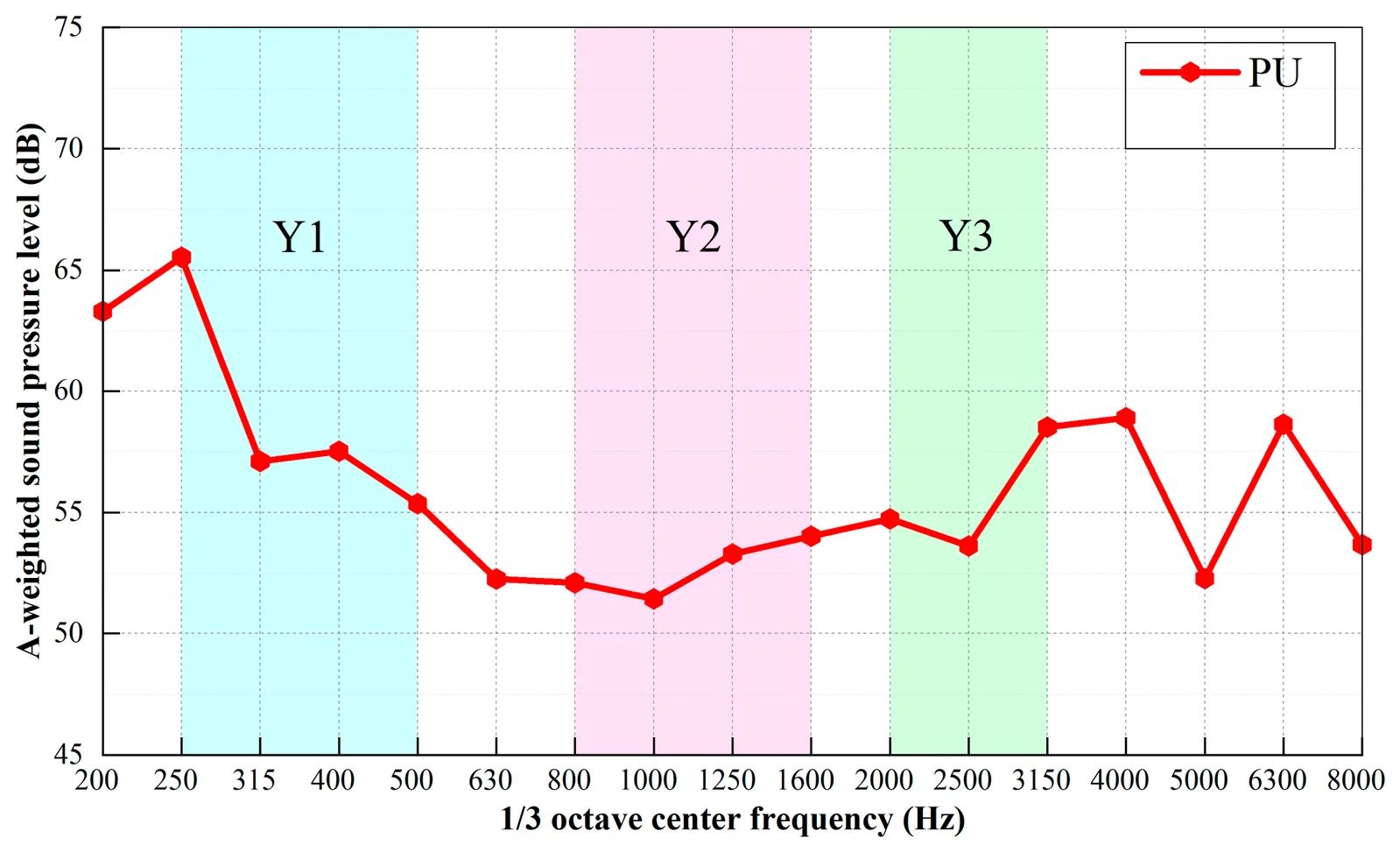
A-weighted SPL spectrum of the driver’s acoustic cavity with the PU acoustic package.

The PU porous acoustic package was applied to the cab model, and the coverage and placement regions are shown in Figure 3. The resulting A-weighted SPL response of the driver’s acoustic cavity is shown in Figure 4. The results show that the PU porous material reduces the cab SPL in the mid- and high-frequency ranges, especially in frequency bands dominated by viscous and thermal dissipation. However, its control capability remains insufficient in the low- and low-to-mid-frequency ranges.

This low-frequency limitation arises from two aspects. First, low-frequency sound waves have relatively long wavelengths, while the thickness of vehicle acoustic packages is constrained by installation space; therefore, velocity gradients and pore-scale dissipation cannot be fully developed inside the material. Second, low-frequency noise in commercial vehicle cabs is often coupled with panel vibration and cavity modes, making it difficult to eliminate local peaks using only pore-based sound absorption. Consequently, a functional structure with low-frequency local-resonance capability is required on the basis of the porous absorbing layer.

## 3. Design of the Six-Cavity Inner–Outer Sector Helmholtz Acoustic Metamaterial

### 3.1. Helmholtz Resonance Mechanism

A Helmholtz resonator consists of a neck channel and a backing cavity, and its acoustic behavior can be approximated as a mass–spring–damper system. The air column in the neck acts as an acoustic mass, the compressible air inside the cavity acts as an acoustic compliance, and the viscous and thermal losses in the neck boundary layer provide the primary damping. When the incident sound frequency approaches the resonance frequency of the structure, the neck air column undergoes strong reciprocating motion. Acoustic energy is exchanged between the neck and the cavity and is dissipated through thermoviscous effects, leading to an absorption peak.

The typical resonance frequency of a Helmholtz resonator can be approximated by

(1) $$f=\frac{c}{2\pi }\sqrt{\frac{S}{VL_{eff}}}$$

where f is the resonance frequency, c is the speed of sound in air, S is the neck cross-sectional area, V is the cavity volume, and $L_{eff}$ is the effective neck length including end correction. This equation indicates that increasing the cavity volume or effective neck length decreases the resonance frequency, whereas increasing the neck cross-sectional area increases the resonance frequency. Therefore, the target low-frequency peaks can be tuned by adjusting the neck dimensions and cavity volume.

### 3.2. COMSOL Model of the Embedded-Neck Single-Cavity Helmholtz Resonator

**Figure 5:**
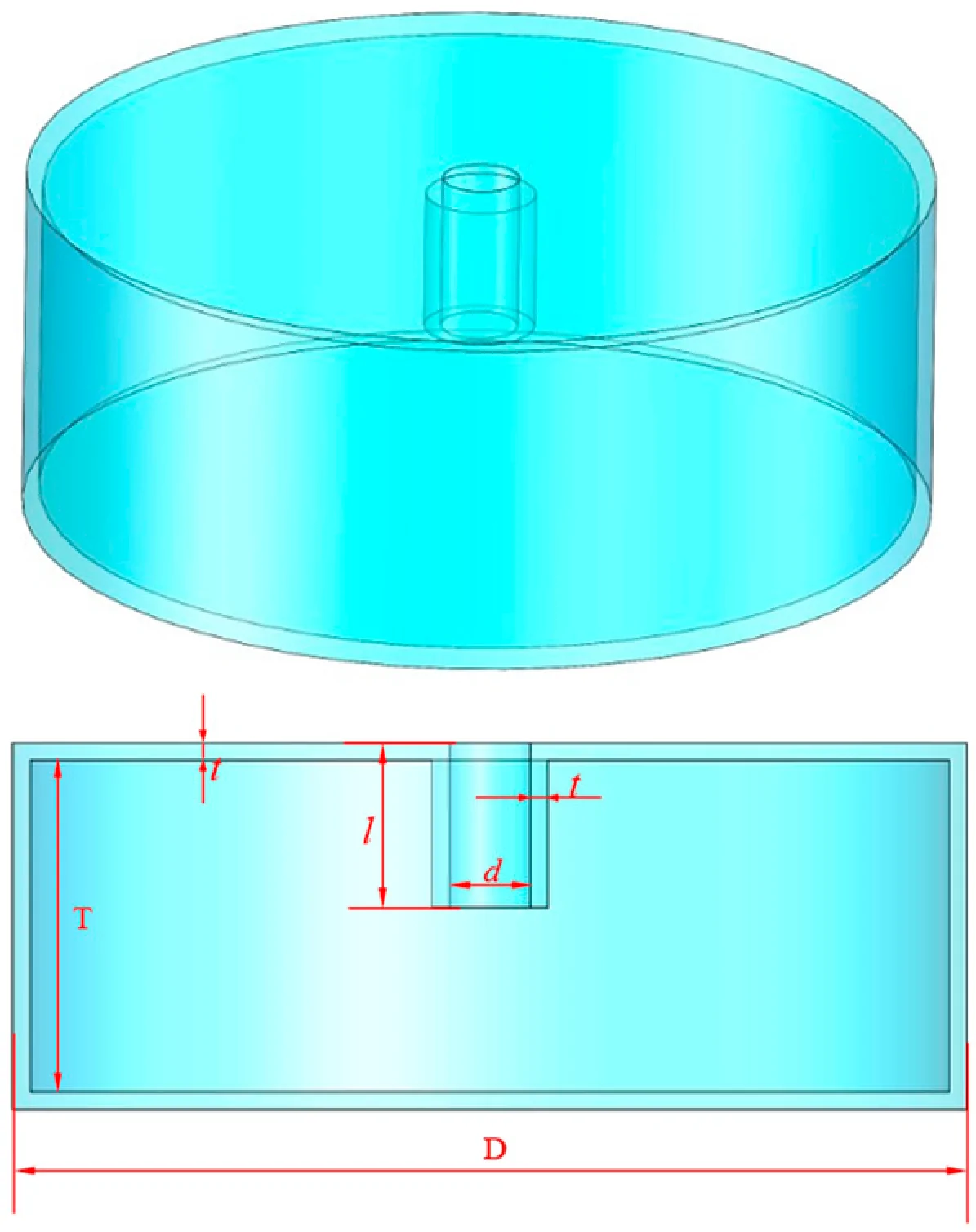
Structural parameters of the embedded-neck single-cavity Helmholtz resonator.

Before designing the six-cavity structure, an embedded-neck single-cavity Helmholtz resonator was first established. Its structural parameters are illustrated in Figure 5. Since normal incidence directly reflects the intrinsic resonant absorption characteristics of the structure, a two-dimensional impedance-tube–resonator coupled model was built in COMSOL, and the transfer-function method was used for numerical post-processing. This procedure does not require direct extraction of the surface impedance. Instead, it determines the reflection characteristics from the complex sound pressures at two measurement points in the impedance tube, thereby obtaining the normal-incidence absorption coefficient. This approach facilitates subsequent parametric scans and keeps the evaluation criterion consistent. The ambient temperature was set to 298.15 K, and the COMSOL parameter settings are listed in Table 2.

Based on this single-cavity model, the neck radius r, cavity height H, and neck depth l were selected as the main parameters for parametric simulation. These parameters correspond to the neck cross-sectional area, backing-cavity volume, and effective neck length, respectively, and are therefore the key structural variables determining the resonance frequency, absorption peak, and peak shape. One-factor-at-a-time scans were performed to obtain the frequency-tuning rules of the single-cavity structure and to provide design guidance for parameter allocation in the subsequent six-cavity inner–outer sector configuration.

**Figure 6:**
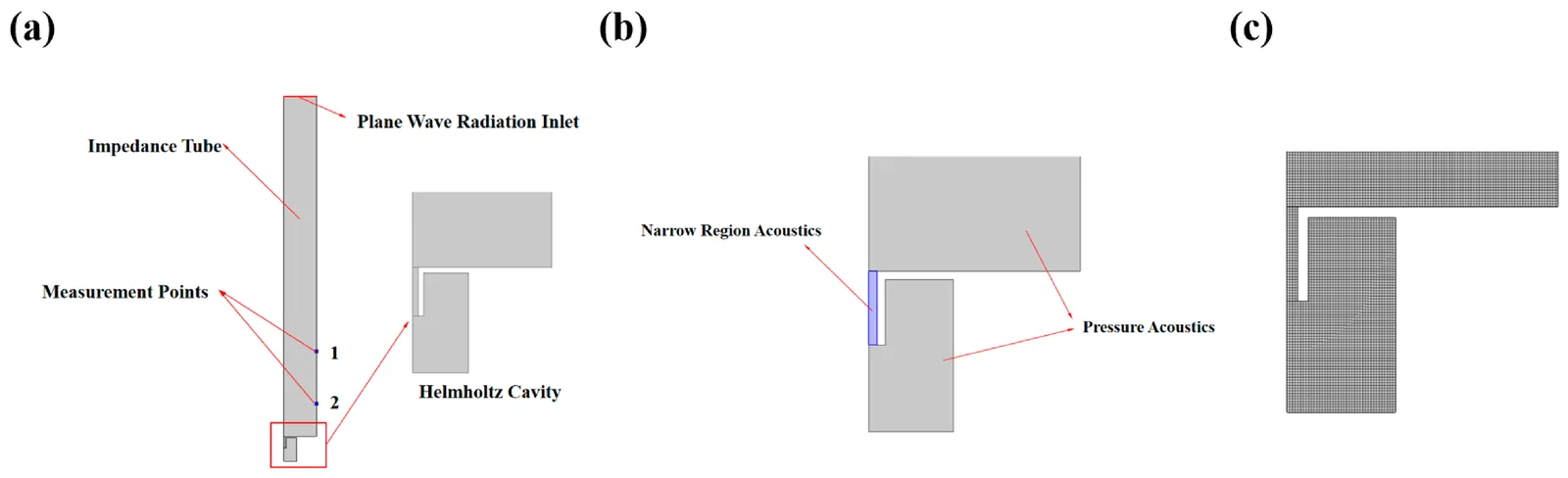
Numerical model of the embedded-neck single-cavity Helmholtz resonator: (**a**) two-dimensional impedance-tube model; (**b**) partitioning of the pressure acoustics and narrow-region acoustics domains; (**c**) mesh distribution of the single-cavity model.

The numerical model of the embedded-neck single-cavity Helmholtz resonator is shown in Figure 6. Figure 6a presents the two-dimensional impedance-tube model, Figure 6b shows the partitioning of the pressure acoustics and narrow-region acoustics domains, and Figure 6c gives the mesh distribution of the single-cavity model.

Let $p_{1}\left(\omega \right)$ and $p_{2}\left(\omega \right)$ denote the complex sound pressures at measurement points 1 and 2, respectively, as shown in Figure 6a. The two-point transfer function is defined as

(2) $$H_{12}(\omega )=\frac{p_{2}}{p_{1}}$$

Within the transfer-function framework, the reflection coefficient r(ω) can be obtained from $H_{12}(\omega )$, the microphone spacing, and the distance from the microphone to the specimen surface. The normal-incidence absorption coefficient is then calculated as

(3) $$\alpha (\omega )=1-{\left|r(\omega )\right|}^{2}$$

This post-processing chain converts the calculated complex sound-pressure field into a physically meaningful absorption coefficient, enabling consistent comparison among different structural parameters.

The main propagation domain of the impedance tube and the cavity domain were solved using the pressure acoustics interface to obtain the steady-state complex pressure field p(x,ω) under frequency-domain excitation. The main acoustic dissipation of the embedded-neck structure is concentrated in the narrow neck and its adjacent connection regions. Because the neck has a small cross-section and strong boundary-layer effects, directly resolving the viscous and thermal boundary layers near the neck wall with conventional pressure acoustics would require extremely fine meshes and would substantially increase the computational cost of the parametric scans.

To address this issue, a Narrow-Region Acoustics treatment was introduced in the neck channel to incorporate viscous and thermal losses induced by thin boundary layers through equivalent impedance or equivalent propagation characteristics. The partitioning between the pressure acoustics domain and narrow-region acoustics domain is shown in Figure 6b. This treatment improves numerical stability and yields a more realistic prediction of peak amplitude and bandwidth, especially when small neck radii are scanned.

For reproducibility, the solid walls of the neck channels in the narrow-region acoustics domain were treated as rigid and no-slip boundaries. Thermal exchange at the wall was modeled using an isothermal-wall assumption, which is suitable for representing thermal boundary-layer losses in narrow air-filled channels connected to a rigid polymer skeleton. The remaining large air domains were solved by pressure acoustics with sound-hard wall boundaries.

The mesh in the propagation domain was generated according to the minimum-wavelength criterion. At the highest calculation frequency $f_{max}$, the minimum wavelength in air is $\lambda_{\min}=c_{0}/f_{\max}$. To suppress numerical dispersion and phase errors, the maximum element size was controlled within $\lambda_{\min}/\left(6\sim 10\right)$, as shown in Figure 6c. In addition, local mesh refinement was applied near the neck opening, cavity corners, and abrupt cross-sectional transitions, where the pressure and velocity gradients are more pronounced. This refinement reduces the amplification of local numerical errors in the overall reflection-coefficient calculation. For the narrow-region acoustics domain, at least two to three elements were ensured across the width of the neck channel, and further refinement was applied at the neck–tube and neck–cavity junctions.

To avoid the potential risk that excessive mesh refinement may lead to a drift in the predicted results, a mesh-independence study was performed using three mesh levels: coarse, medium, and fine. The peak absorption frequency $f_{p}$, the maximum absorption coefficient $\alpha_{max}$, and the bandwidth-related indicators were compared among the three mesh levels. When the variations in these key indicators were below the prescribed threshold, the solution was considered mesh-independent. Once the baseline mesh passed the mesh-independence check, the subsequent variations in the absorption curves could be attributed primarily to changes in structural parameters rather than discretization errors.

The baseline parameters used in the impedance tube simulation are defined as follows: the impedance tube radius is denoted by $R_{1}$, the tube length by $L_{3}$, the distance between the two measurement points by $L_{2}$, the neck radius of the embedded tube by $r_{2}$, the embedded depth or neck length by l, the cavity height by T, the cavity radius by $r_{1}$, and the cavity wall thickness by $t_{1}$. The parameters used for the 200 Hz design case are listed in Table 3.

**Figure 7:**
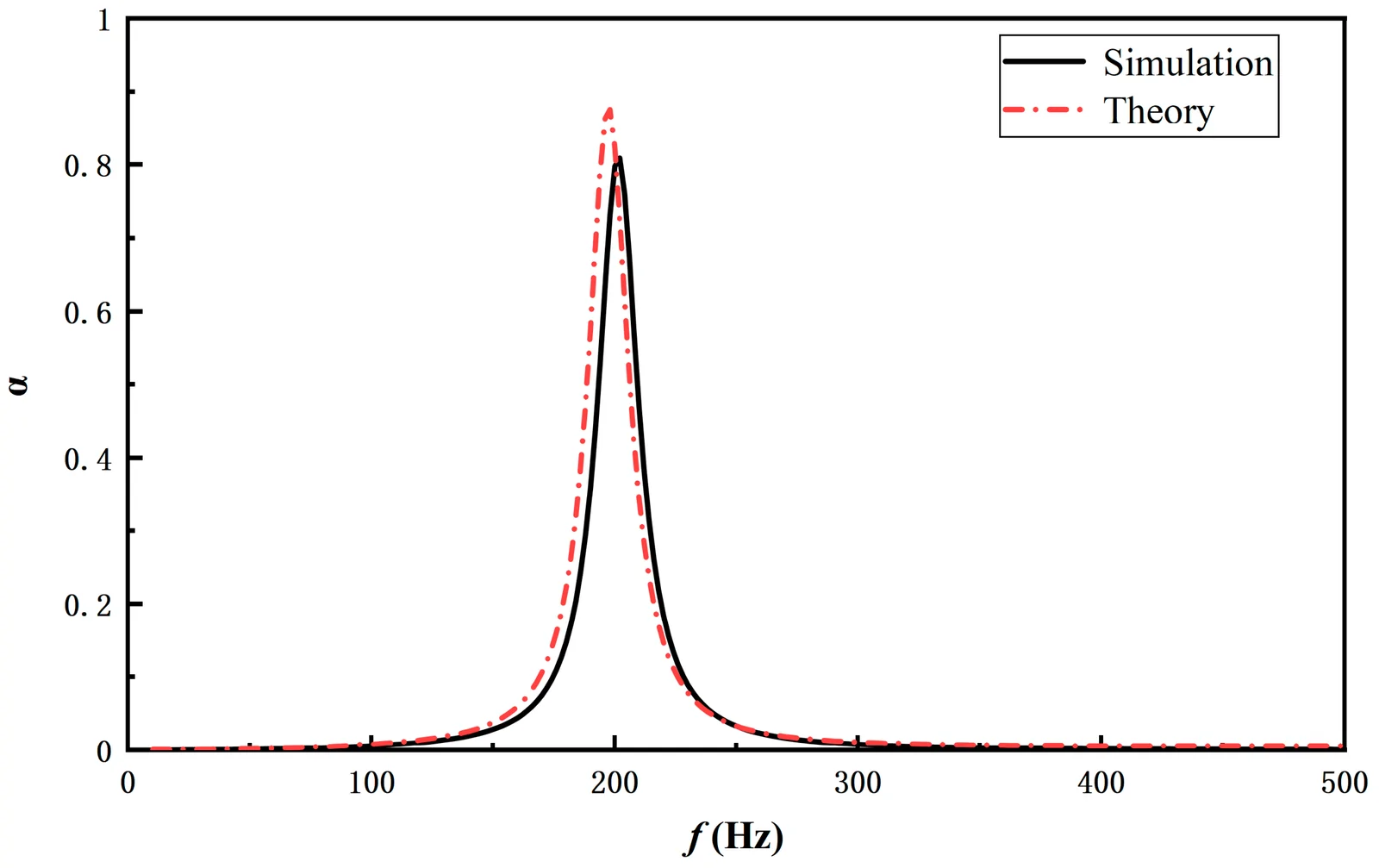
Comparison between the theoretical and simulated results for the 200 Hz resonator.

The simulated and theoretical results are compared in Figure 7. The peak frequency obtained from the COMSOL simulation agrees well with the theoretical prediction. Because the numerical model accounts for thermoviscous losses in the neck and local impedance effects, the simulated peak absorption coefficient is slightly lower than that predicted by the ideal theoretical model. Nevertheless, the peak-frequency deviation remains within an acceptable range, indicating that the model is suitable for subsequent parametric scans.

### 3.3. Parametric Analysis of the Single-Cavity Structure

#### 3.3.1. Effect of Neck Radius $r_{2}$ on Absorption Performance

**Figure 8:**
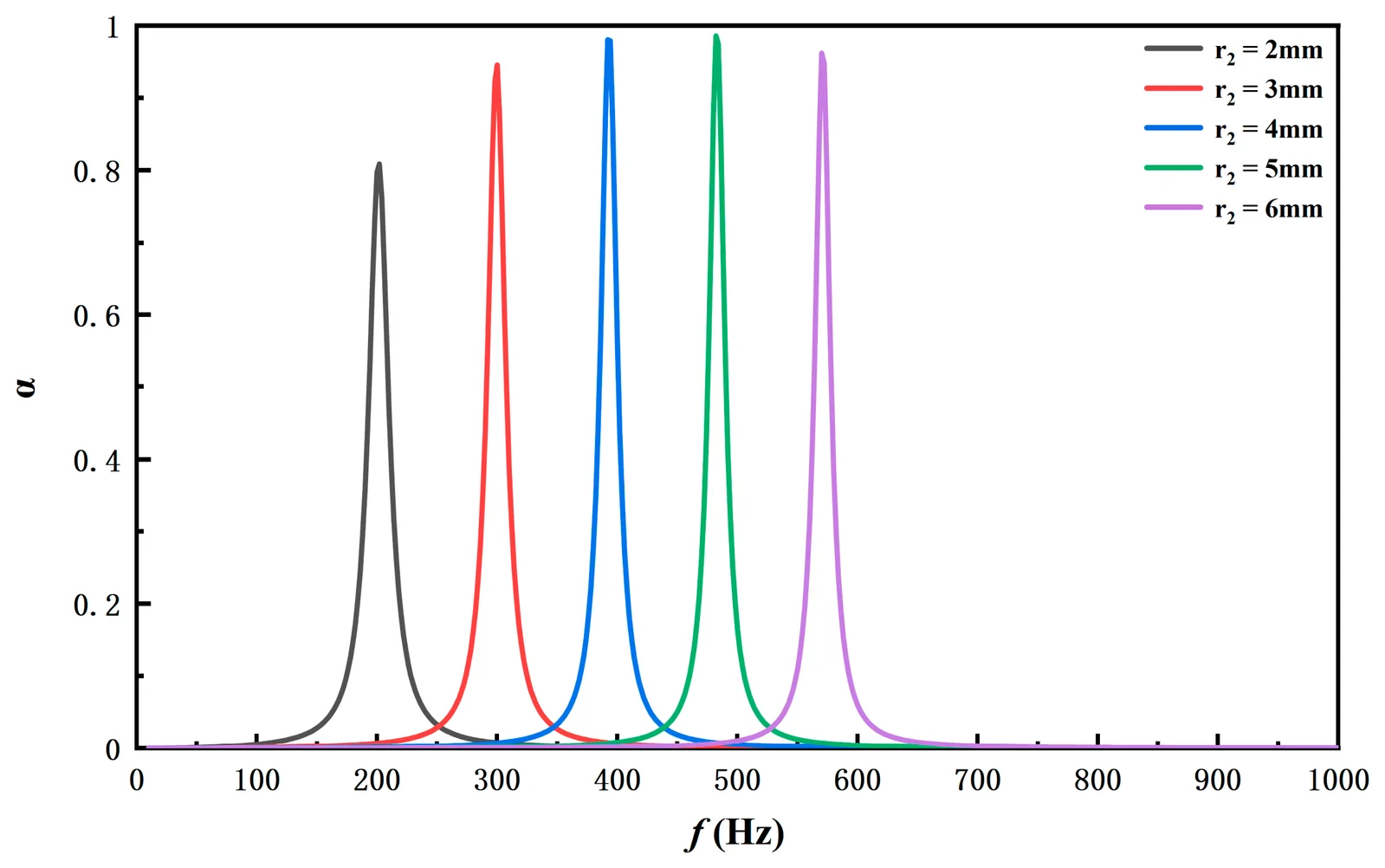
Absorption coefficient curves for different neck radii.

With the cavity geometry and neck depth l kept constant, the neck radius $r_{2}$ was scanned, and the corresponding absorption coefficient curves are shown in Figure 8. The trend is clear: as $r_{2}$ increases, the resonance peak shifts toward higher frequencies. Meanwhile, the peak amplitude increases and the peak shape becomes sharper.

This peak shift can be directly explained by the frequency relation. When V and $L_{eff}$ are approximately unchanged, increasing $r_{2}$ increases the neck cross-sectional area S, which shifts the resonant frequency upward. The simultaneous changes in peak amplitude and bandwidth are associated with the scale-dependent thermoviscous losses in the neck. For a smaller neck radius, the boundary layer occupies a larger proportion of the cross-section, leading to stronger viscous shear and heat exchange, higher equivalent resistance, a lower peak amplitude, and a wider peak. As $r_{2}$ increases, the relative thermoviscous loss decreases, and the system approaches a stronger resonant absorption state, resulting in a higher and sharper peak.

#### 3.3.2. Effect of Cavity Height T on Absorption Performance

**Figure 9:**
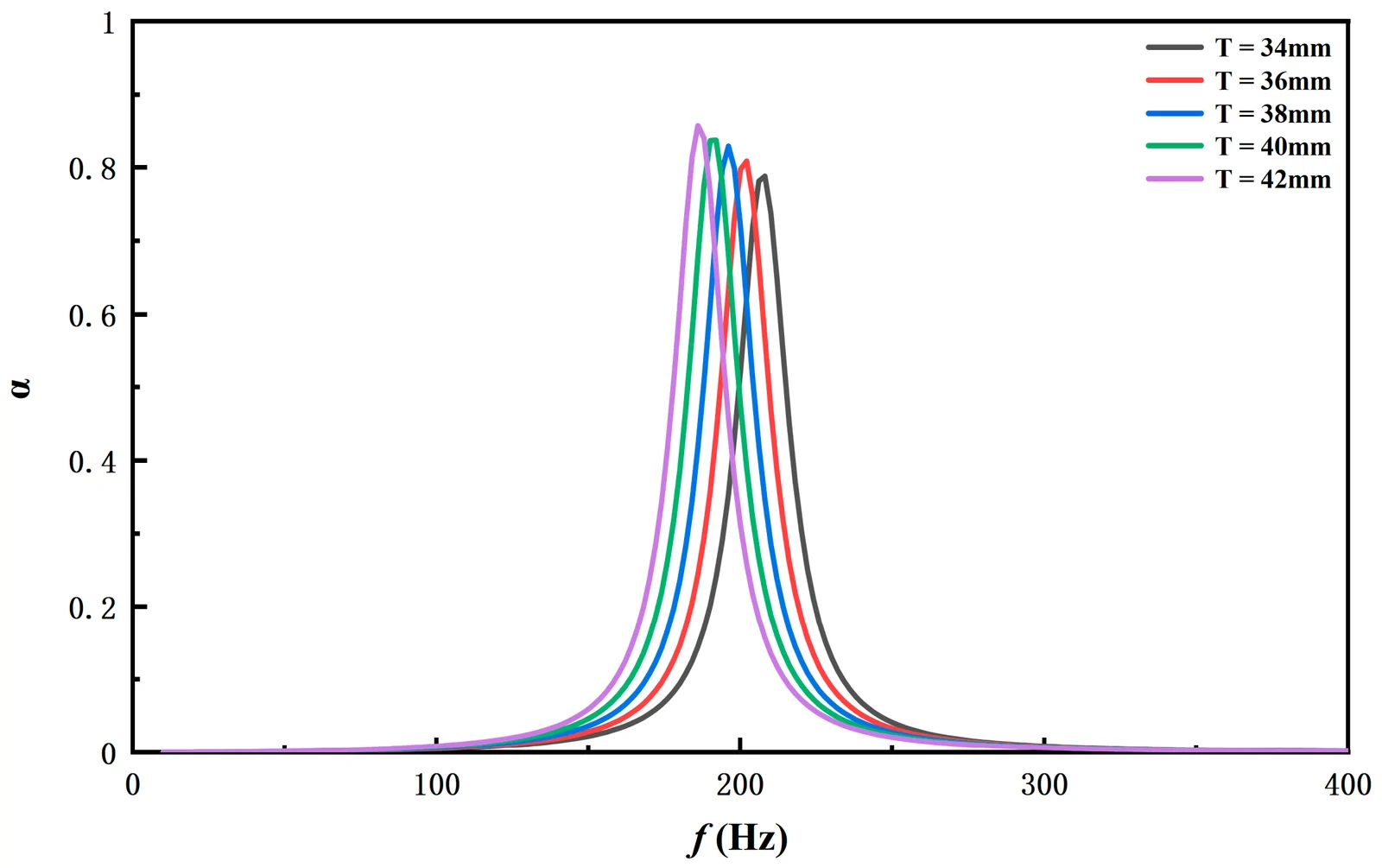
Absorption coefficient curves for different cavity heights.

With the neck geometry kept unchanged, the cavity height T was scanned, and the absorption coefficient curves are shown in Figure 9. Compared with the strong sensitivity of the neck radius, the effect of T is more moderate. As T increases, the resonance peak shifts toward lower frequencies, while the overall peak amplitude changes only slightly.

For an approximately cylindrical cavity, the cavity volume V is proportional to T. Therefore, increasing T increases the acoustic compliance of the cavity and consequently decreases the resonant frequency. The peak amplitude is less sensitive to T than to $r_{2}$ because the dominant thermoviscous dissipation occurs mainly in the neck and its adjacent region, whereas the cavity volume primarily affects the acoustic energy storage. When T is relatively small, the cavity becomes thinner, and the local velocity distribution inside the cavity may be enhanced, leading to minor variations in peak amplitude and bandwidth.

#### 3.3.3. Effect of Neck Depth l on Absorption Performance

**Figure 10:**
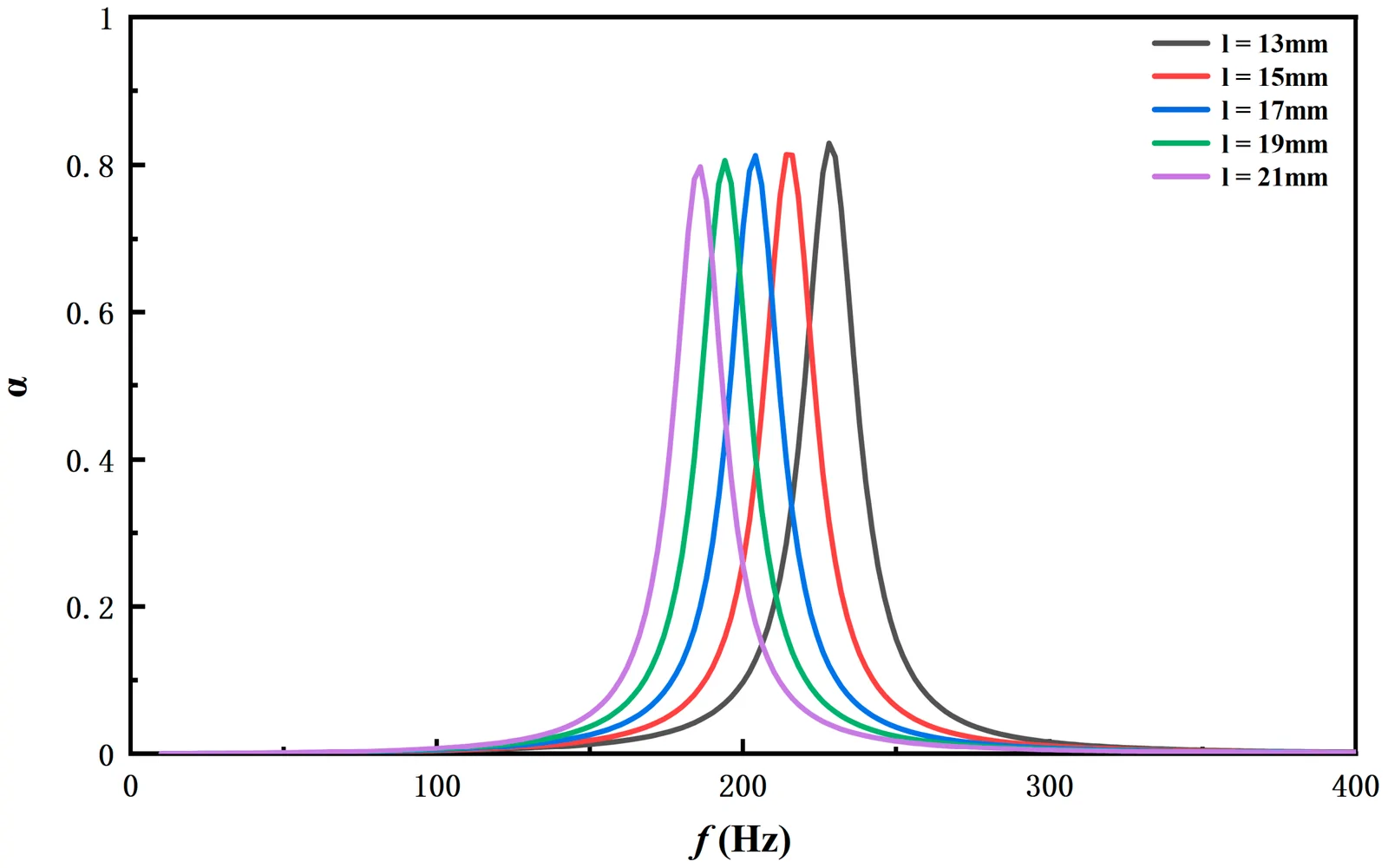
Absorption coefficient curves for different neck depths.

With the neck radius and cavity geometry kept constant, the neck depth l was scanned. The absorption coefficient curves are shown in Figure 10. As l increases, the resonance peak shifts toward lower frequencies, and the peak shape becomes slightly flattened and broadened.

This trend can also be interpreted using the Helmholtz resonance relation. Increasing l increases the effective neck length $L_{eff}$ and thus increases the equivalent acoustic mass, resulting in a lower resonant frequency. In addition, the neck–cavity end boundary of the embedded-neck structure is relatively complex, and the loss associated with l is more easily amplified. A longer neck also increases the accumulated viscous and thermal losses along the channel, which may slightly reduce the peak amplitude and broaden the absorption peak, corresponding to a lower quality factor.

Overall, the peak position and peak shape of the embedded-neck Helmholtz resonator are mainly governed by the neck cross-sectional area S, cavity volume V, and effective neck length $L_{eff}$. The neck radius $r_{2}$ directly controls the rapid migration of the resonant frequency by changing S, and it also significantly affects the peak amplitude and sharpness because thermoviscous losses are highly sensitive to the channel scale. The cavity height T mainly changes V, resulting in a relatively smooth low-frequency shift of the peak. The neck depth l shifts the peak toward lower frequencies by increasing the effective neck length $L_{eff}$, while the accumulated losses may broaden the peak.

### 3.4. Design of the Six-Cavity Helmholtz Acoustic Metamaterial

To achieve effective absorption coverage over the 200–630 Hz low-to-mid-frequency range and to obtain multiple controllable resonance peaks within a limited thickness, a spatial partitioning and cavity grouping strategy was adopted. Multiple Helmholtz resonant cavities with different geometric parameters were arranged inside a single metamaterial unit so that different sub-cavities resonate near their target frequencies, generating a multi-peak absorption response in the overall frequency domain. Acoustically, the multi-cavity structure behaves as a parallel-coupled system, and its equivalent input impedance is jointly determined by the input impedances of the sub-cavities. When a sub-cavity approaches its own resonance, its acoustic admittance increases significantly. The superposition of multiple admittance peaks makes the overall impedance more likely to approach the characteristic impedance of air at multiple frequencies, thereby producing multi-peak absorption.

Considering the specimen size of a 100 mm impedance tube and the thickness constraints of vehicle acoustic packages, the outer diameter and total thickness of the metamaterial were set to 100 mm and 30 mm, respectively. The top plate, bottom plate, outer wall, and internal partition walls were all set to 2 mm. A six-cavity parallel unit consisting of three outer low-frequency cavities and three inner mid-frequency cavities was constructed. This dimensional system enables six target resonance peaks at 200, 250, 315, 400, 500, and 630 Hz to be realized with manufacturable hole diameters and neck lengths, and provides consistent geometric input for subsequent COMSOL impedance-tube simulations.

#### 3.4.1. Design Objective and Structural Configuration

A single Helmholtz resonator naturally exhibits a narrow single-peak response. Although its peak position can be tuned by the neck and cavity volume, it is difficult to maintain multiple high absorption peaks within 200–630 Hz using a single cavity. Considering that practical low-to-mid-frequency noise spectra often exhibit concentrated low-frequency energy and require compensation between low-frequency peaks, the target resonances were divided into two groups: an outer ring group at 200, 250, and 315 Hz, and an inner ring group at 400, 500, and 630 Hz. The design strategy is to assign the low-frequency peaks to larger cavities and longer necks, while the mid-frequency peaks are assigned to smaller cavities with short necks and variable apertures, thereby realizing six controllable resonance peaks within the limited thickness.

The target frequencies of the six-cavity Helmholtz metamaterial were selected according to the residual low-frequency and low-to-mid-frequency noise characteristics after applying the PU porous acoustic package, rather than by targeting a single isolated acoustic mode. The purpose of the proposed composite acoustic package is to achieve broadband noise reduction by compensating for the frequency bands in which the PU porous material is inherently limited. Therefore, the six target frequencies were arranged within 200–630 Hz to cover the typical residual low-frequency bands that cannot be further optimized effectively by the PU porous material alone.

The circular unit was circumferentially divided into three sectors, and each sector was further separated radially into an outer cavity and an inner cavity by an annular partition. The outer cavities have larger volumes and are used for the 200/250/315 Hz low-frequency group, whereas the inner cavities have smaller volumes and are used for the 400/500/630 Hz mid-frequency group. This arrangement reduces the need for extreme parameter adjustment: low-frequency tuning is primarily achieved by larger volume and longer neck length, whereas mid-frequency tuning is primarily achieved by fixed short necks and variable hole diameters.

The outer low-frequency cavities adopt a uniform aperture and use different neck lengths to align with 200, 250, and 315 Hz. At low frequencies, tuning by excessively reducing the aperture would increase viscous resistance and reduce the peak amplitude; therefore, a uniform aperture was selected to improve manufacturability and reduce frequency deviation caused by aperture error. For the inner mid-frequency cavities, the neck length is provided by the top-plate thickness and fixed at 2 mm, and the hole diameter is back-calculated to tune the resonances to 400, 500, and 630 Hz. This short-aperture strategy is more reasonable because additional neck tubes would reduce the effective inner-cavity volume and increase structural complexity.

#### 3.4.2. Target Frequencies and Sub-Cavity Parameter Design

Under approximately normal incidence, the openings of all cavities are subjected to the same acoustic pressure. For the j-th sub-cavity, the input impedance is defined as

(4) $$Z_{j}=\frac{p}{U_{j}}$$

where *p* is the acoustic pressure at the opening and $U_{j}$ is the volume velocity. For a parallel multi-cavity structure, the total volume velocity is

(5) $$U_{\mathrm{tot}}=\sum_{j=1}^{6}U_{j}$$

and the total input impedance satisfies

(6) $$\frac{1}{Z_{\mathrm{tot}}}=\sum_{j=1}^{6}\frac{1}{Z_{j}}$$

Equation (6) explains the origin of multi-peak absorption: each sub-cavity contributes a large admittance near its own resonance, and the superposition of several admittance peaks makes the overall impedance closer to the characteristic impedance of air at multiple frequencies.

The resonant frequency of a single Helmholtz cavity is written as

(7) $$f_{0}=\frac{c_{0}}{2\pi }\sqrt{\frac{S}{VL_{\mathrm{eff}}}}$$

where $c_{0}$ is the speed of sound in air, S is the aperture area, V is the backing-cavity volume, and $L_{eff}$ is the effective neck length including the end correction. For a circular aperture,

(8) $$S=\frac{\pi d^{2}}{4}$$

and the effective neck length is expressed as

(9) $$L_{\mathrm{eff}}=l+\kappa d$$

where l is the physical neck length and κd represents the equivalent end correction. Because the end correction depends on the boundary condition, aperture thickness, and confinement at the cavity side, different correction coefficients were used for the outer long-neck cavities and the inner short-aperture cavities.

It should be noted that the empirical end-correction coefficient used above provides an initial analytical estimate. In practice, 3D-printing tolerances, layer-wise surface roughness, aperture roundness, and slight deviations in neck length may change the actual equivalent end correction and therefore shift the resonance frequency relative to the ideal analytical prediction [35,36].

For the outer-ring low-frequency cavities, the aperture diameter was fixed at d = 4 mm, and the physical neck length l was adjusted to tune the target frequencies. Rearranging Equation (7) gives the required effective length:

(10) $$L_{\mathrm{eff}}=\frac{S}{V{\left(\frac{2\pi f_{t}}{c_{0}}\right)}^{2}}$$

For the outer long-neck structure, the effective length model is

(11) $$L_{\mathrm{eff}}=l+\kappa_{\mathrm{out}}d,\;\kappa_{\mathrm{out}}=0.85$$

and the physical neck length is therefore

(12) $$l=L_{\mathrm{eff}}-0.85d$$

The calculated effective and physical neck lengths of the three outer-ring cavities are listed in Table 4. The 200 Hz cavity requires the longest neck. If a straight neck cannot be accommodated in the available space, a folded neck can be used to maintain the required equivalent centerline length, while avoiding sharp bends and sudden contractions.

For the inner-ring mid-frequency cavities, the cavity volume is smaller. If the resonance were tuned by increasing the neck length, the effective volume would be reduced and the manufacturing complexity would increase. Therefore, the inner ring adopts a short-aperture form with the neck length fixed by the 2 mm top-plate thickness. The aperture diameter is then calculated from Equations (7)–(9) to match the target frequencies of 400, 500, and 630 Hz.

#### 3.4.3. Final Structural Scheme

**Figure 11:**
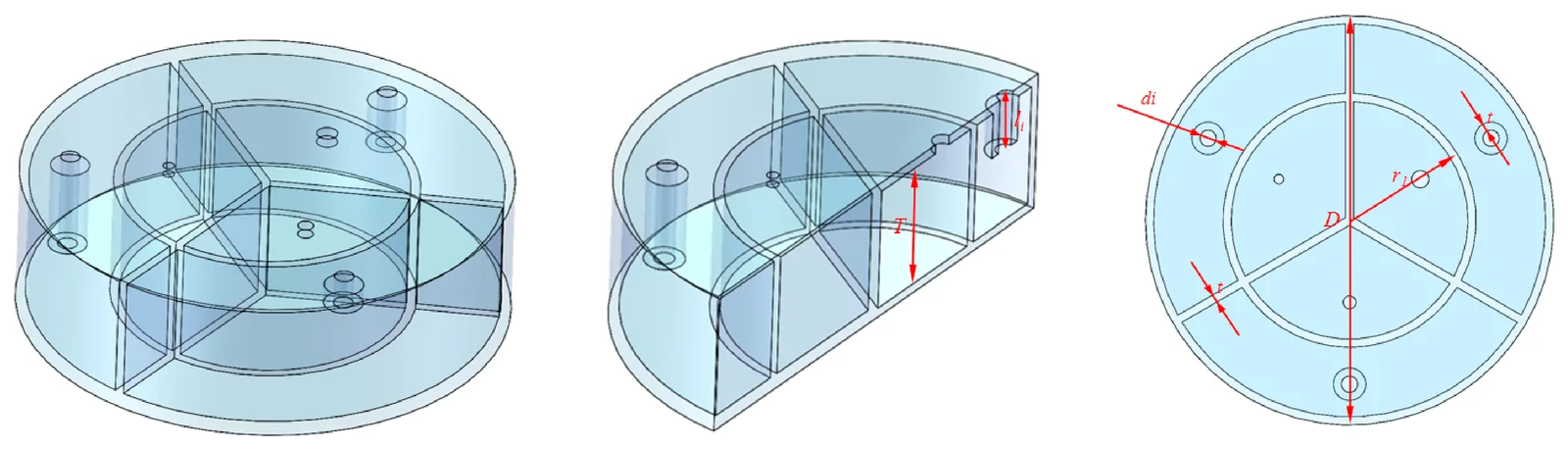
Three-dimensional view of the six-cavity inner–outer sector Helmholtz resonator.

The final structural parameters are summarized in Table 5 and Table 6, and the three-dimensional view of the six-cavity structure is shown in Figure 11.

### 3.5. COMSOL Simulation of the Six-Cavity Helmholtz Acoustic Metamaterial

To verify the absorption coverage of the proposed metamaterial unit within the target frequency range and to further reveal the local resonance and dissipation mechanism of the parallel multi-cavity structure, a three-dimensional impedance-tube–specimen coupled numerical model was established in COMSOL. The normal-incidence absorption coefficient of the metamaterial was solved in the frequency domain, and particle velocity distributions at typical peak frequencies were extracted for mechanism analysis.

**Figure 12:**
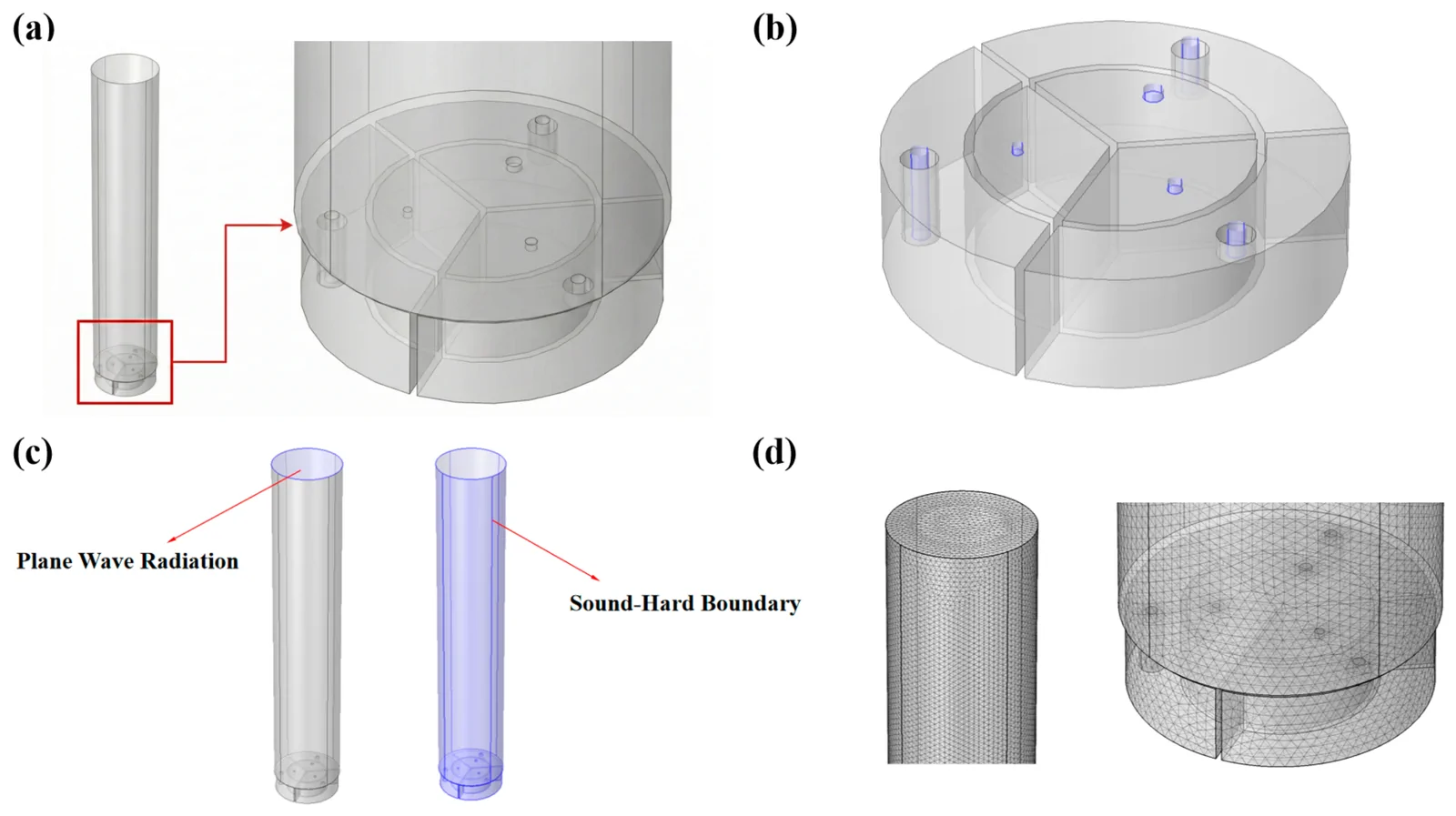
Numerical setup of the three-dimensional impedance-tube model for the six-cavity Helmholtz metamaterial: (**a**) coupled impedance-tube–metamaterial model; (**b**) narrow-region acoustics domains in the neck channels; (**c**) plane-wave radiation and sound-hard boundary settings; (**d**) mesh distribution of the impedance tube and locally refined metamaterial unit.

The three-dimensional impedance-tube model for the six-cavity Helmholtz metamaterial is shown in Figure 12. Figure 12a presents the coupled impedance-tube–metamaterial model, while Figure 12b shows the narrow-region acoustics domains assigned to the neck channels. The impedance tube provides the plane-wave propagation and reflection environment. The internal cavities, necks, and narrow channels of the metamaterial were modeled using the real three-dimensional geometry to capture non-axisymmetric effects, inter-cavity coupling paths, and dissipation dominated by local boundary layers. The metamaterial skeleton was treated as a rigid boundary in this section.

To balance geometric accuracy and the representation of losses in narrow channels, the main impedance-tube domain and the larger cavities of the metamaterial were modeled using pressure acoustics, while the neck and slit channels were treated using narrow-region acoustics to incorporate viscous and thermal losses more reasonably. This avoids excessive mesh refinement near the walls of extremely narrow structures.

The incident excitation and boundary conditions in the three-dimensional impedance-tube model were set to be consistent with the experimental impedance-tube configuration. As shown in Figure 12c, a plane-wave radiation or port excitation was imposed at the upstream end of the tube, with the wave propagating along the tube axis. A unit-amplitude incident pressure of 1 Pa was used, and the port cross-section was kept consistent with the tube cross-section to generate approximately pure plane-wave incidence. The tube wall and the skeleton walls of the sample were set as sound-hard boundaries, assuming a well-sealed structure.

Compared with two-dimensional or axisymmetric models, the three-dimensional simulation is more sensitive to mesh quality. Therefore, a mesh strategy combining global wavelength control, local refinement, and critical-channel control was adopted. In the impedance tube and main cavity regions, the mesh size satisfied the discretization requirement associated with the minimum wavelength at the highest frequency. Local refinement was applied to the necks, slits, sharp corners, partition ends, and cavity-connection regions, as shown in Figure 12d. These regions determine the resonance peak position and amplitude, and an overly coarse mesh may cause peak-frequency shifts or numerical attenuation of the peak amplitude. When narrow-region acoustics is used, the extreme mesh requirements inside the narrow channels can be moderately relaxed while maintaining the validity of the interface. The frequency range of the solver was set to 10–1000 Hz with a step of 2 Hz.

**Figure 13:**
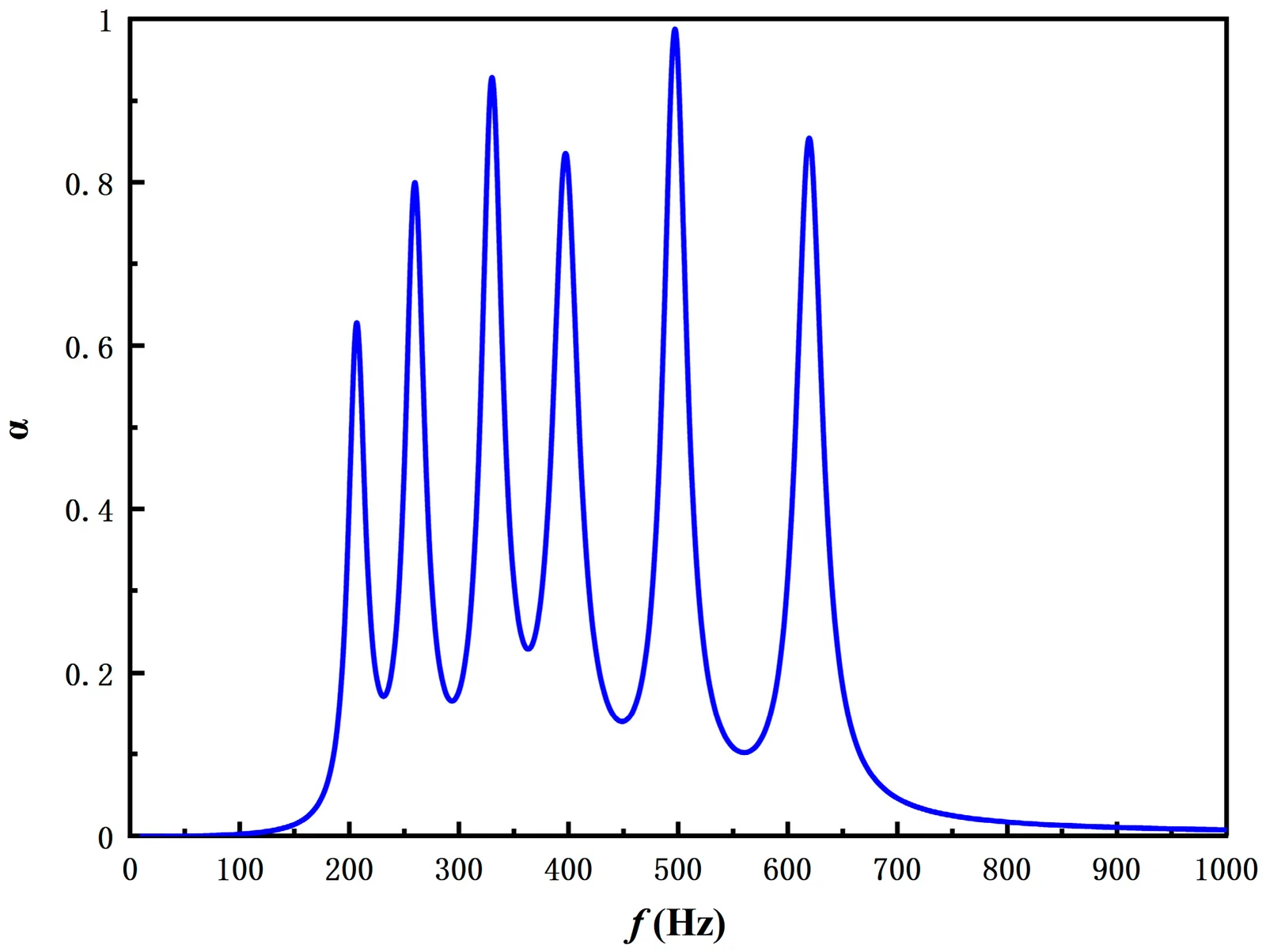
Absorption coefficient of the six-cavity metamaterial.

After the three-dimensional frequency-domain solution was completed, the reflection coefficient was calculated using the impedance-tube post-processing method described above, and the normal-incidence absorption coefficient was obtained. For the multi-cavity Helmholtz metamaterial, different sub-cavities exhibit local resonance at different frequencies, leading to multiple absorption peaks within the target band. As shown in Figure 13, the structure forms absorption peaks near 200, 250, 315, 400, 500, and 630 Hz, indicating that the outer low-frequency cavities and inner mid-frequency cavities respond near the preset target frequencies.

**Figure 14:**
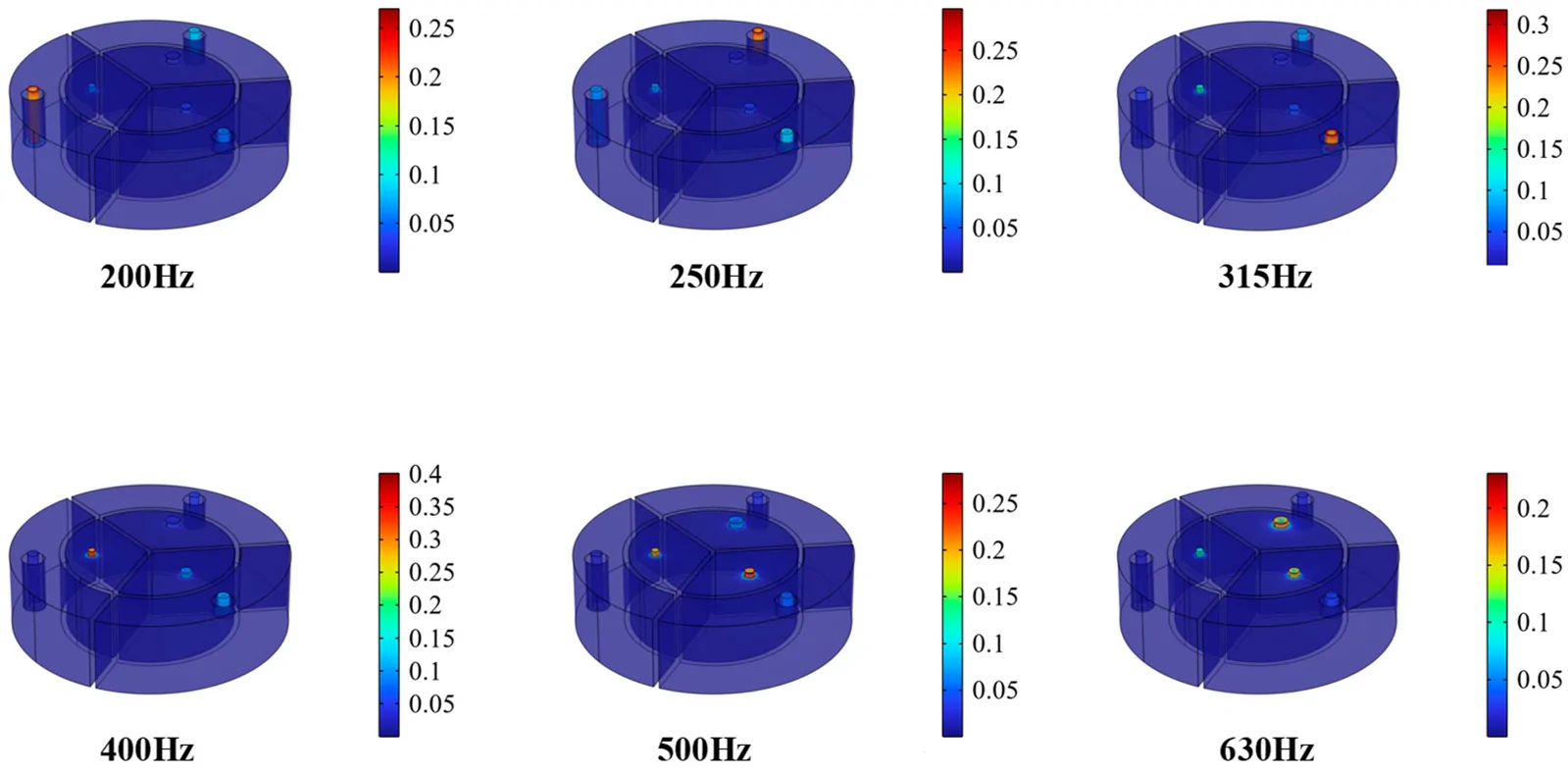
Particle velocity distributions at typical target frequencies.

To reveal the local resonance mechanism responsible for the absorption peaks, particle velocity distributions were extracted at representative peak frequencies. Figure 14 shows that, at different target frequencies, the high-velocity regions are mainly concentrated in the necks or neck–cavity junctions of the corresponding sub-cavities. This indicates that the relevant sub-cavities are selectively excited and generate local resonant dissipation. The result further verifies the rationality of the six-cavity parameter design and the functional division of the outer low-frequency and inner mid-frequency cavities.

## 4. Synergistic Noise Reduction in the PU–Six-Cavity Helmholtz Metamaterial Composite Acoustic Package

### 4.1. Simulation of the Synergistic Absorption Coefficient of the Helmholtz Metamaterial and PU Layer

#### 4.1.1. Composite Configuration, Physics Domains, and Parameter Settings

To evaluate the absorption performance after coupling the Helmholtz acoustic metamaterial with the PU material, a three-dimensional impedance-tube model of the composite acoustic package was further established based on the modeling method described above. The normal-incidence absorption coefficient of the composite structure was calculated.

The composite acoustic package consists of two components. The first is the multi-cavity Helmholtz acoustic metamaterial designed in the previous section, which mainly enhances low-frequency resonant absorption. The second is the PU foam absorbing layer, which provides effective broadband porous dissipation in the mid- and high-frequency ranges. The two layers are stacked along the direction of incident sound propagation to form an integrated composite structure. The metamaterial layer preferentially generates local resonance in response to low-frequency sound waves, whereas the sound transmitted into the rear PU porous layer is further attenuated through viscous and thermal losses in the interconnected pores. This forms a synergistic absorption mechanism combining low-frequency resonant enhancement and mid-to-high-frequency porous dissipation.

**Figure 15:**
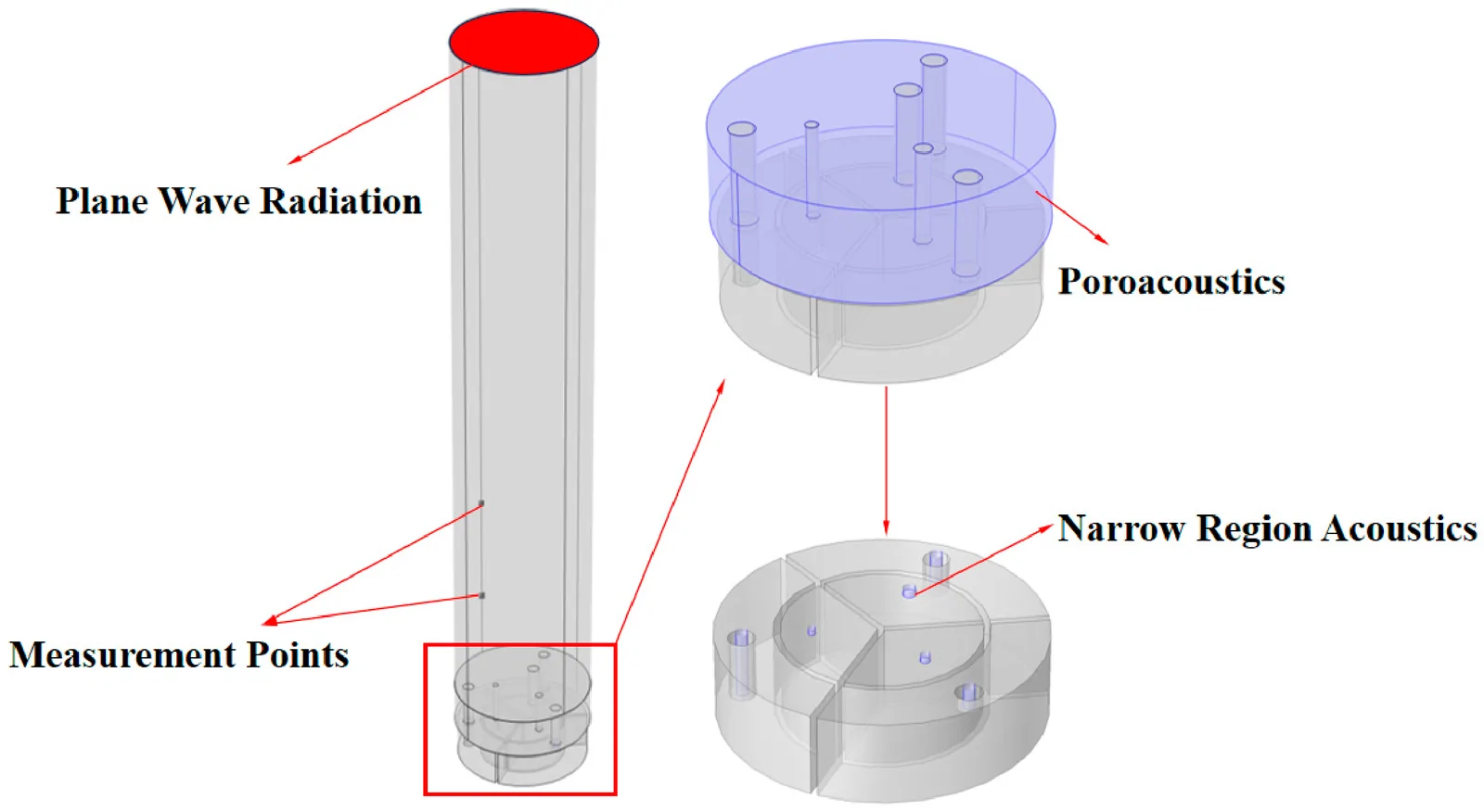
Impedance-tube model and physics-domain partitioning for the composite acoustic package.

From the modeling perspective, the Helmholtz metamaterial is a typical locally resonant absorber, in which thermoviscous boundary-layer effects in the narrow necks strongly affect the absorption peak positions and amplitudes. The PU porous layer, by contrast, is an equivalent-fluid porous medium whose acoustic characteristics can be described by effective density and effective bulk modulus. Therefore, different physics domains were applied in COMSOL: narrow-region acoustics was used in the metamaterial neck regions to represent boundary-layer losses, while the PU porous layer was modeled as an equivalent fluid using the Johnson–Champoux–Allard model. The remaining air domains in the impedance tube and larger cavities were solved using pressure acoustics. The domain partitioning is shown in Figure 15. Plane-wave radiation was imposed at the incident end, the side walls were set as sound-hard boundaries, and the rigid backing plate at the end of the structure was also defined as a rigid boundary. The frequency range was set according to the test range of the 100 mm impedance tube, with the low-frequency enhancement region and the effective PU absorption region being the primary focus.

**Figure 16:**
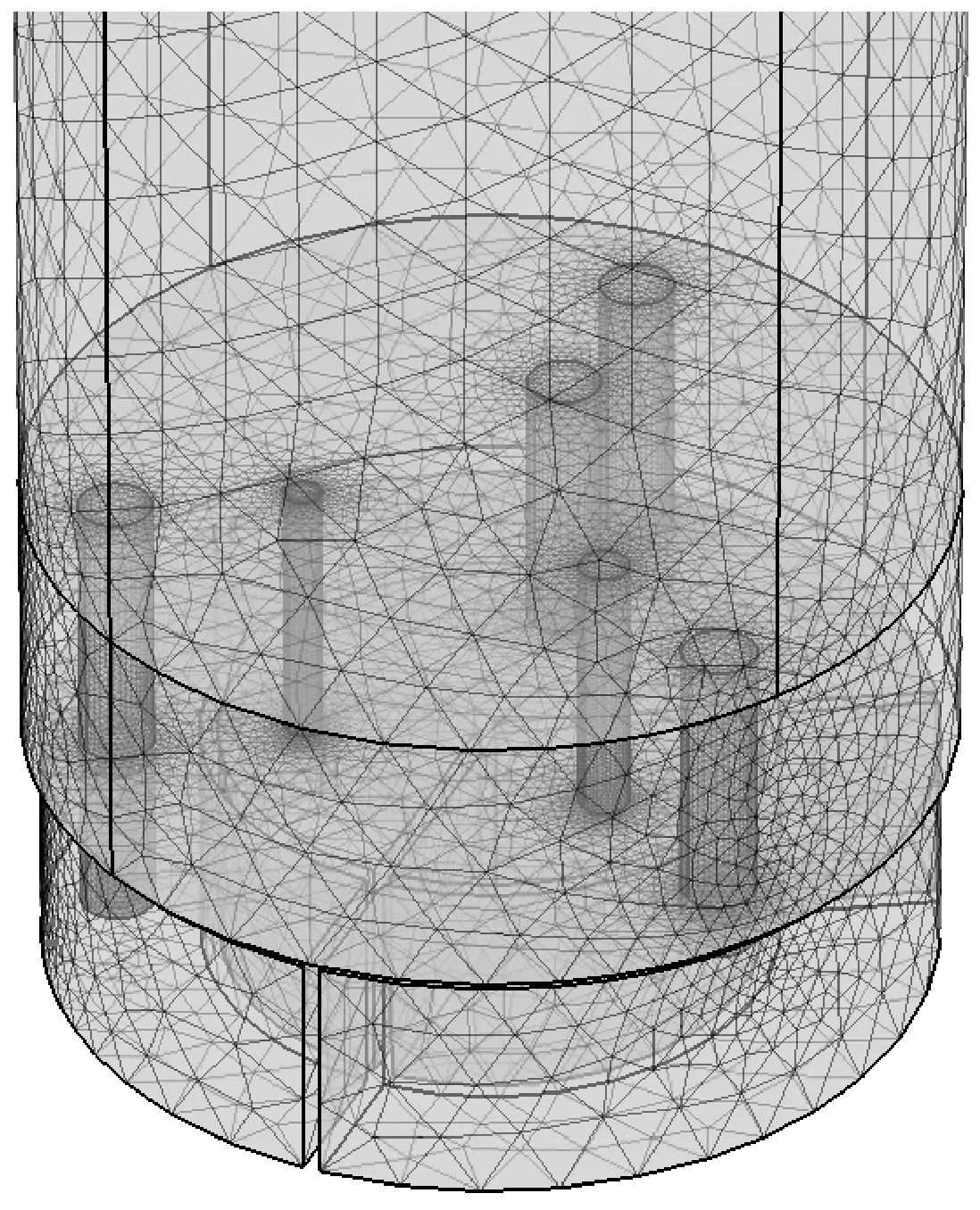
Mesh distribution of the composite acoustic package.

The model used a circular impedance-tube cross-section with a diameter of 100 mm to maintain consistency with the subsequent Brüel & Kjær experimental platform. The composite structure was mounted in front of the rigid backing at the end of the impedance tube. The surface pressure and normal velocity response of the composite structure were solved under plane-wave excitation, from which the normalized surface acoustic impedance, reflection coefficient, and absorption coefficient were calculated. Since the model contains a large-scale air domain, complex local cavities, and narrow-neck structures, the mesh was designed to balance global wave resolution and local boundary-layer accuracy. The air domain and large cavities were meshed using free tetrahedral elements controlled by the minimum wavelength, while the narrow necks were locally refined. The PU porous layer was meshed more uniformly because it was described as an equivalent medium, as shown in Figure 16.

#### 4.1.2. Simulation Results of the Synergistic Absorption Coefficient

**Figure 17:**
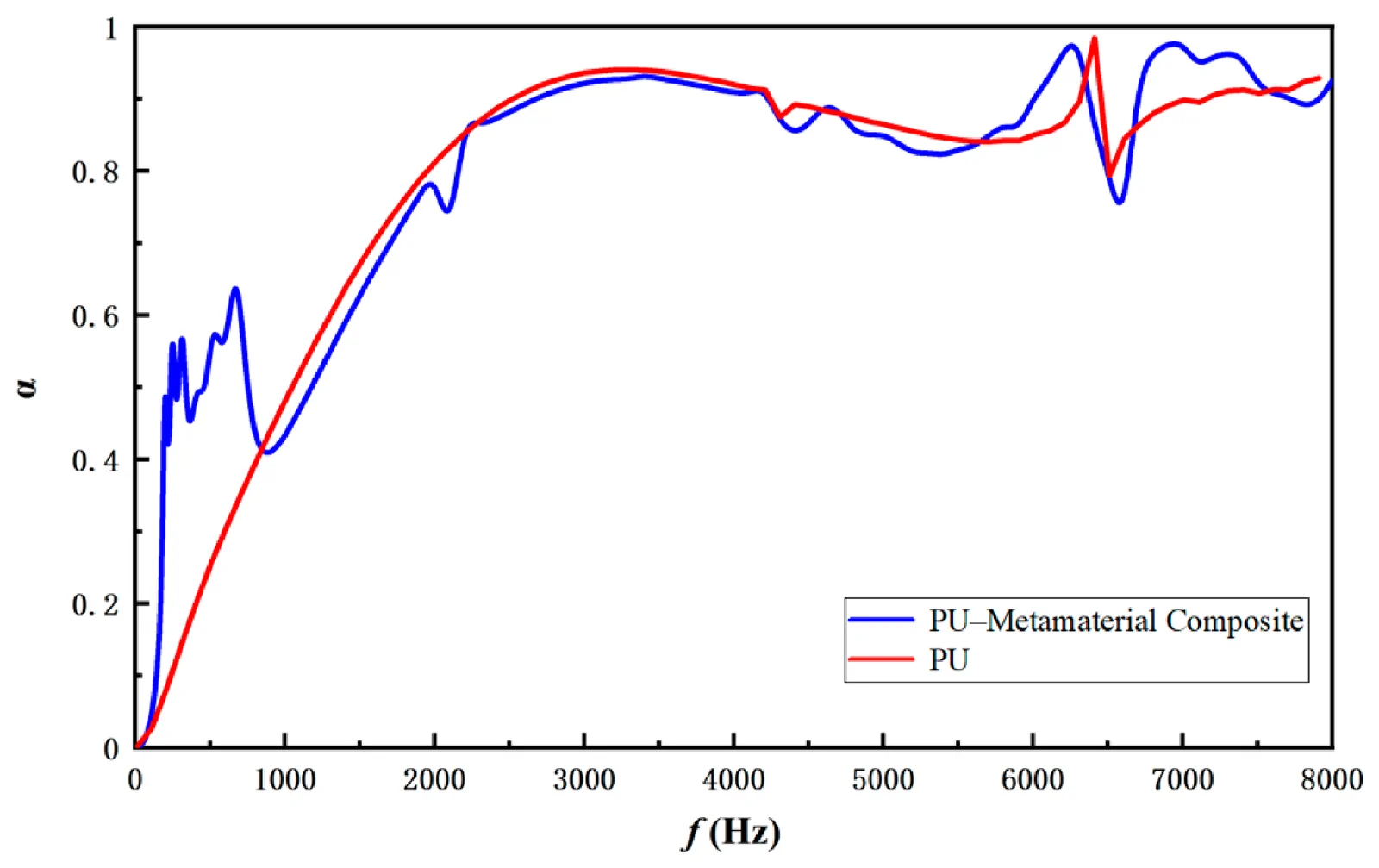
Comparison of absorption coefficient curves for the PU porous layer and PU–six-cavity Helmholtz metamaterial composite structure.

Figure 17 compares the simulated normal-incidence absorption coefficients of the PU porous layer and the PU–six-cavity Helmholtz metamaterial composite structure. Both curves exhibit the general characteristic of low absorption at very low frequencies and increasing absorption toward the mid- and high-frequency ranges. However, in the low-frequency region, the composite structure shows more pronounced local absorption enhancement than the PU layer alone.

Below approximately 1000 Hz, the absorption response of the composite structure differs clearly from that of the PU porous layer. The PU layer exhibits relatively low absorption in this region and increases gradually with frequency, indicating limited dissipation capability for long-wavelength low-frequency sound. In contrast, the PU–six-cavity composite structure shows an earlier rise in absorption and forms distinct local peaks, demonstrating that the front Helmholtz resonant units capture and dissipate low-frequency acoustic energy that is otherwise difficult for the PU layer to absorb. In the range of several hundred hertz, the absorption coefficient of the composite structure is significantly higher than that of the PU layer, confirming the low-frequency enhancement effect of the metamaterial.

In the 1000–2500 Hz range, both curves increase rapidly and gradually converge. In this range, the PU material itself begins to operate effectively, and the viscous and thermal losses induced by its interconnected pores become stronger. The rear PU porous layer in the composite structure continues to provide broadband dissipation, so the overall absorption coefficient increases accordingly. Although the advantage of the composite structure over the PU layer alone becomes less pronounced in this band, the introduction of the metamaterial does not degrade the original mid-frequency absorption capability.

In the higher-frequency range shown in the simulation, the PU layer becomes the dominant absorbing mechanism and both structures maintain relatively high absorption. The difference between the composite structure and the PU layer is small, indicating that the rear PU porous layer governs mid-to-high-frequency dissipation. The local fluctuations observed in the composite curve are related to the internal multi-cavity structure, neck inertia, and interlayer coupling, which affect the equivalent surface impedance. Overall, the composite structure improves low-frequency absorption while maintaining the broadband absorption capability of the PU layer, thereby forming a complementary mechanism rather than a simple structural superposition.

### 4.2. Impedance-Tube Experiment and Simulation Comparison

#### 4.2.1. Experimental Apparatus and Testing Principle

Although COMSOL simulations can reveal the absorption characteristics of the composite acoustic package, factors such as material-parameter dispersion, manufacturing errors, and idealized boundary conditions may lead to discrepancies between simulation and reality. Therefore, impedance-tube experiments were performed to measure the actual absorption performance of the composite structure, and the experimental data were used as the input basis for subsequent system-level simulations.

**Figure 18:**
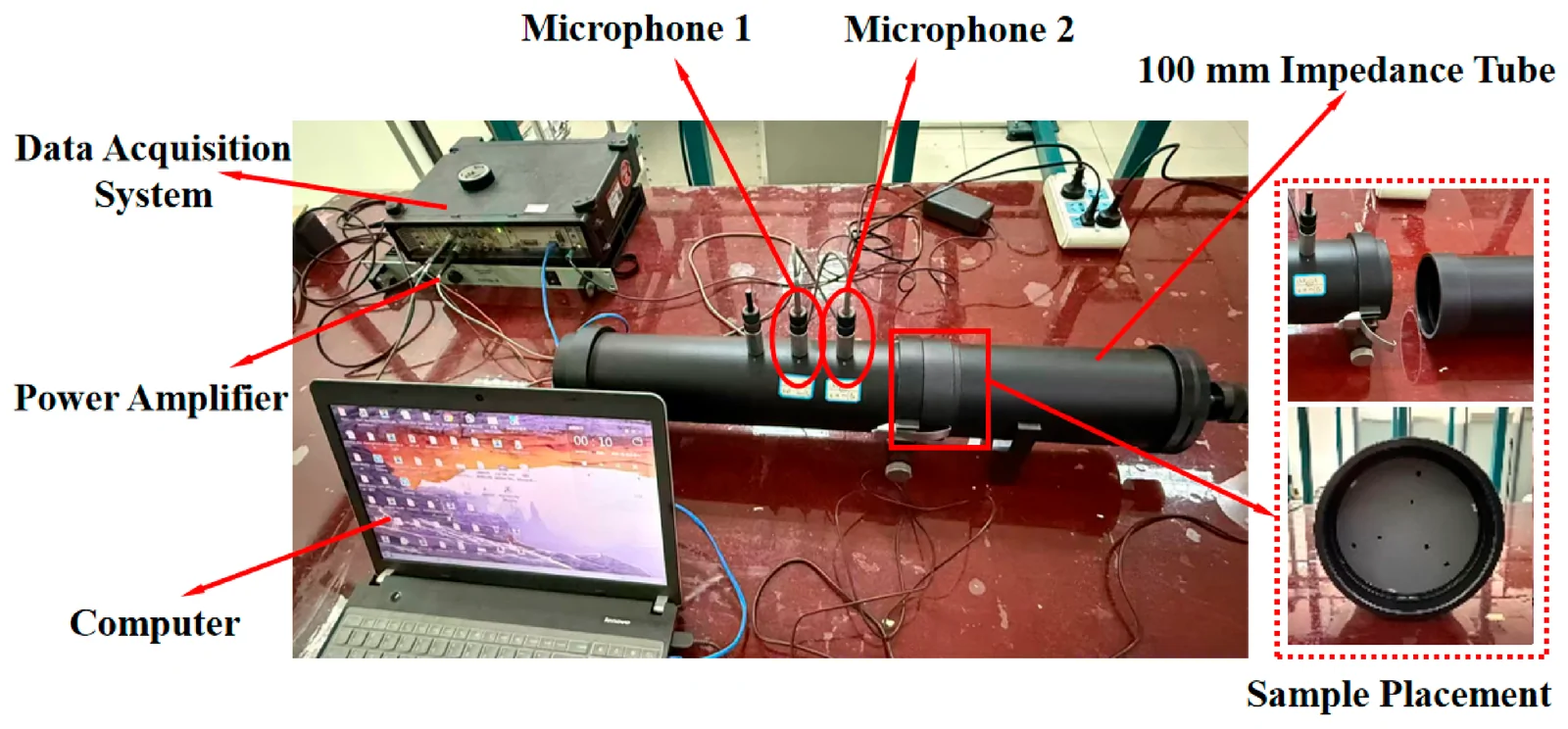
Brüel & Kjær 100 mm impedance-tube test system.

A Brüel & Kjær impedance-tube test system was used to measure the normal-incidence absorption coefficient of the composite acoustic package specimens. The inner diameter of the impedance tube was 100 mm, corresponding to the low-to-mid-frequency range of interest in this study. The system consists of a signal source, power amplifier, data-acquisition unit, impedance tube, front-end acquisition module, and analysis software. In the experiment, the two-microphone transfer-function method was used to obtain the reflection coefficient at the specimen surface, from which the normal-incidence absorption coefficient was calculated. The test system is shown in Figure 18.

The two-microphone method establishes an approximately one-dimensional plane-wave field in the impedance tube. By measuring the sound-pressure transfer function between two fixed microphone positions, the complex reflection coefficient at the material surface can be inverted, and the normal-incidence absorption coefficient can then be calculated. This method is applicable not only to single materials but also to the composite acoustic package studied here.

It should also be noted that the impedance-tube method provides a normal-incidence validation under controlled one-dimensional wave propagation. The acoustic field inside a commercial vehicle cab may contain diffuse-field or random-incidence components due to multiple reflections, structural radiation, and complex source paths. Therefore, the impedance-tube results were used to verify the intrinsic material absorption characteristics, while the engineering applicability was further examined by full-vehicle simulation and in-vehicle testing. Future work will include diffuse-field validation in an alpha cabin or reverberation room to further evaluate the composite package under more realistic acoustic incidence conditions.

#### 4.2.2. Specimen Fabrication and Installation

**Figure 19:**
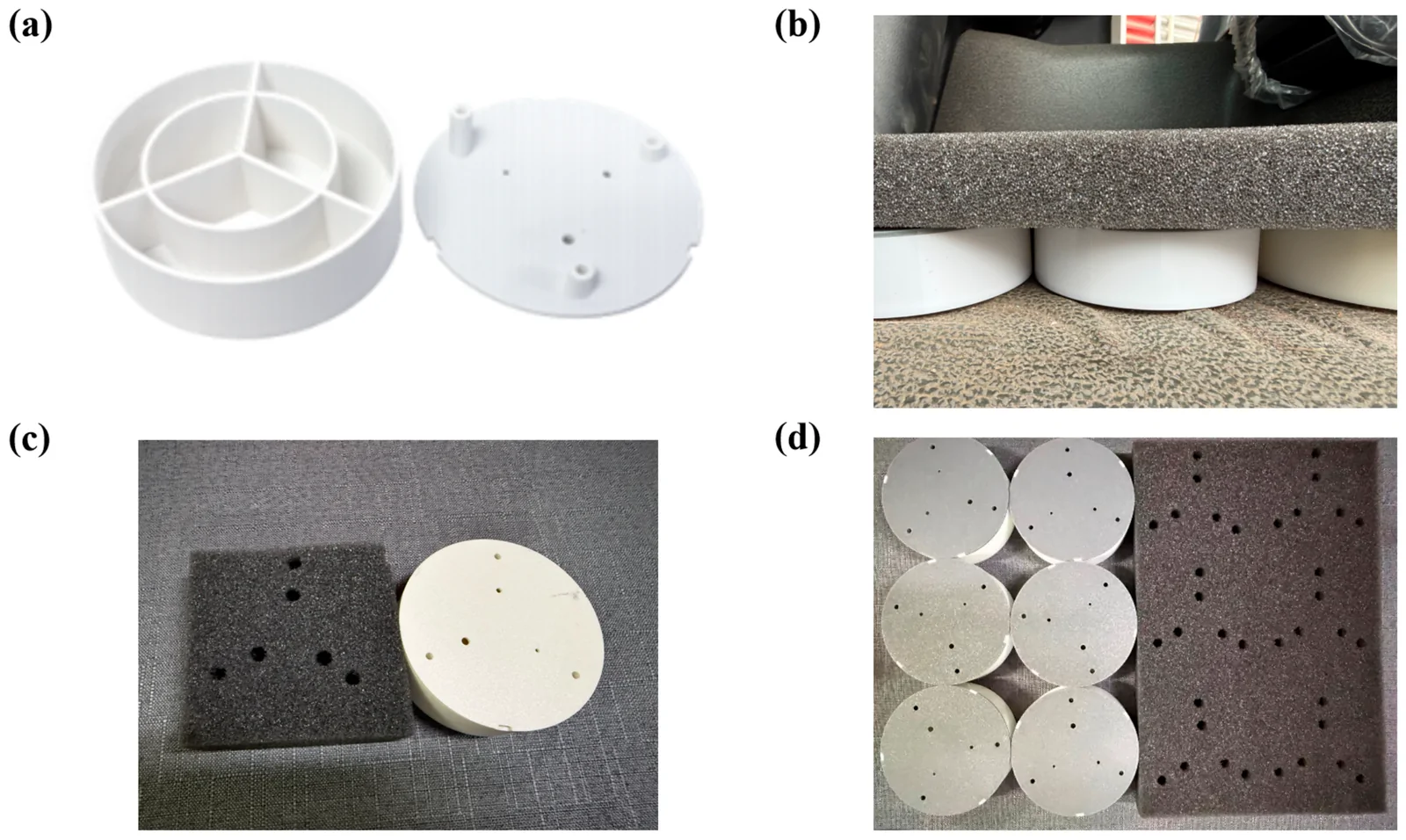
Fabricated specimens of the composite acoustic package: (**a**) six-cavity Helmholtz metamaterial components; (**b**) stacked PU porous layer and metamaterial specimens; (**c**) separated PU layer and metamaterial specimen; (**d**) PU–metamaterial composite specimens.

To ensure comparability between the experiment and simulation, the impedance-tube specimen geometry was kept consistent with the numerical model. The overall specimen diameter was 100 mm to match the tube cross-section. The thicknesses of the metamaterial layer and the PU porous layer were 30 mm and 30 mm, respectively, resulting in a total composite thickness of 60 mm. The metamaterial components were fabricated by 3D printing according to the designed dimensions, and the PU porous layer was cut to match the specimen size. During assembly, the PU porous layer was stacked behind the metamaterial layer along the direction of sound propagation. To minimize the influence of edge leakage, the composite specimen was installed tightly against the inner wall of the impedance tube, and sealing tape or flexible sealing material was used when necessary. The fabricated specimens of the composite acoustic package are shown in Figure 19.

It should be noted that deviations inevitably exist between the actual specimens and the ideal numerical models, including manufacturing errors in the metamaterial neck dimensions, compression deformation of the PU layer, and variations in interlayer contact. These factors can influence the resonance peak positions, peak amplitudes, and high-frequency smoothness of the response. Therefore, the experimental results not only validate the simulation model but also demonstrate the practical feasibility of the composite structure under real fabrication conditions.

#### 4.2.3. Comparison Between Experimental and Simulated Results

**Figure 20:**
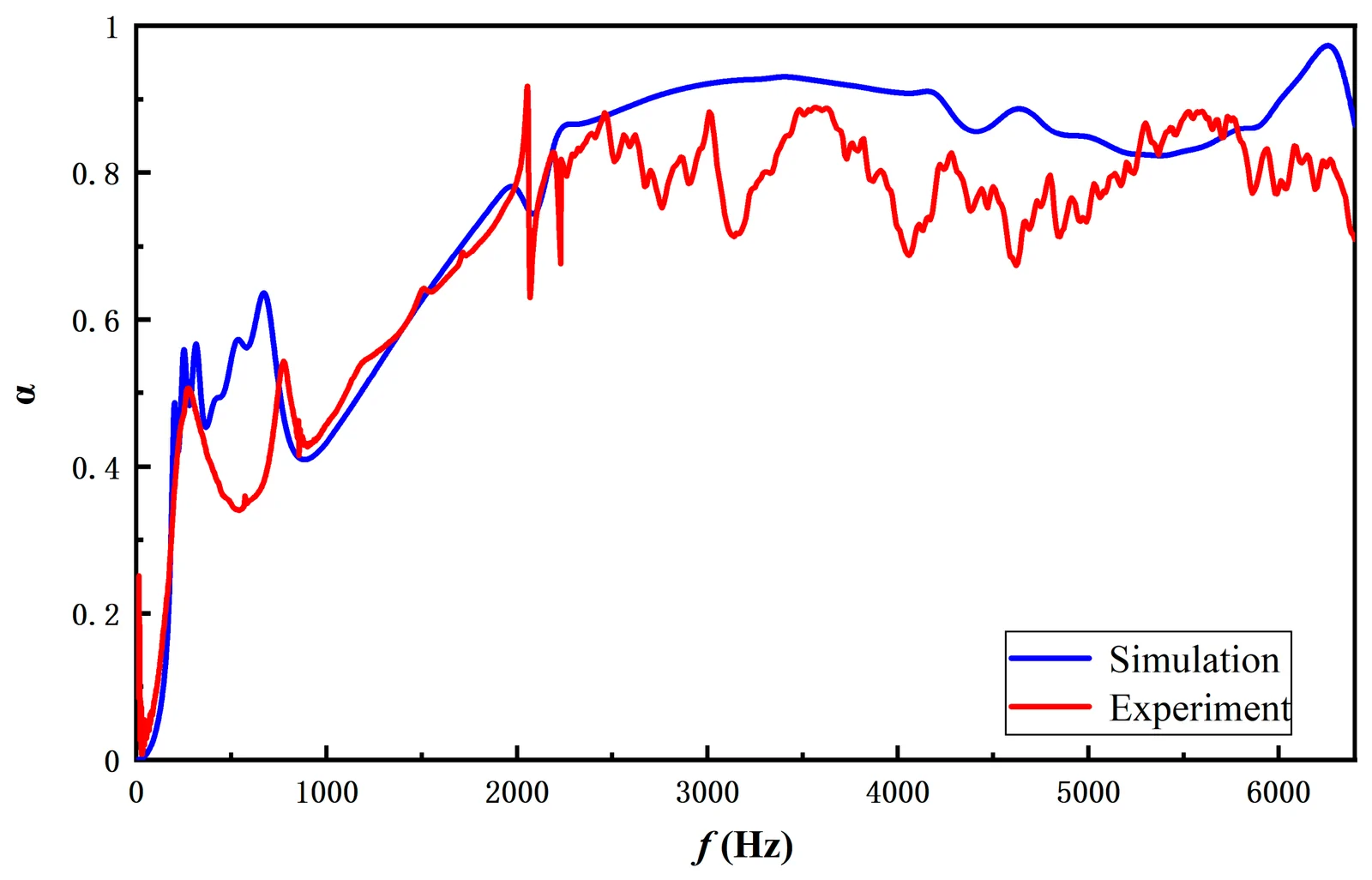
Comparison between experimental and simulated absorption coefficients of the composite acoustic package.

Figure 20 compares the measured absorption coefficient of the composite acoustic package with the COMSOL simulation result. The experimental and simulated curves show generally consistent trends over the main frequency range. Both results exhibit distinct absorption peaks in the low-frequency region, indicating that the Helmholtz resonant units in the composite structure remain effective in the real specimen. In the mid- and high-frequency ranges, the absorption coefficient remains relatively high, confirming the broadband dissipation contribution of the PU porous layer. This agreement demonstrates the rationality of the simulation model and the parameter settings used for the composite structure.

Local discrepancies are still observed between the experimental and simulated curves. First, slight shifts in some resonance frequencies may be caused by dimensional errors of the metamaterial cavities and changes in the actual effective neck length. Second, the experimental peak amplitudes may be slightly lower than the simulated values because surface roughness, imperfect interlayer contact, and edge leakage introduce additional losses. Third, the experimental curve in the mid- and high-frequency ranges may be more fluctuating, which is related to the fitting accuracy of the equivalent PU parameters, internal material non-uniformity, and the limitations of large-diameter impedance tubes at higher frequencies. Overall, the experimental results validate the fundamental design objective of low-frequency enhancement and broadband compatibility, and support the subsequent definition of the composite structure as a new acoustic treatment for system-level simulations.

### 4.3. Full-Vehicle Simulation Validation with the Composite Acoustic Package

**Figure 21:**
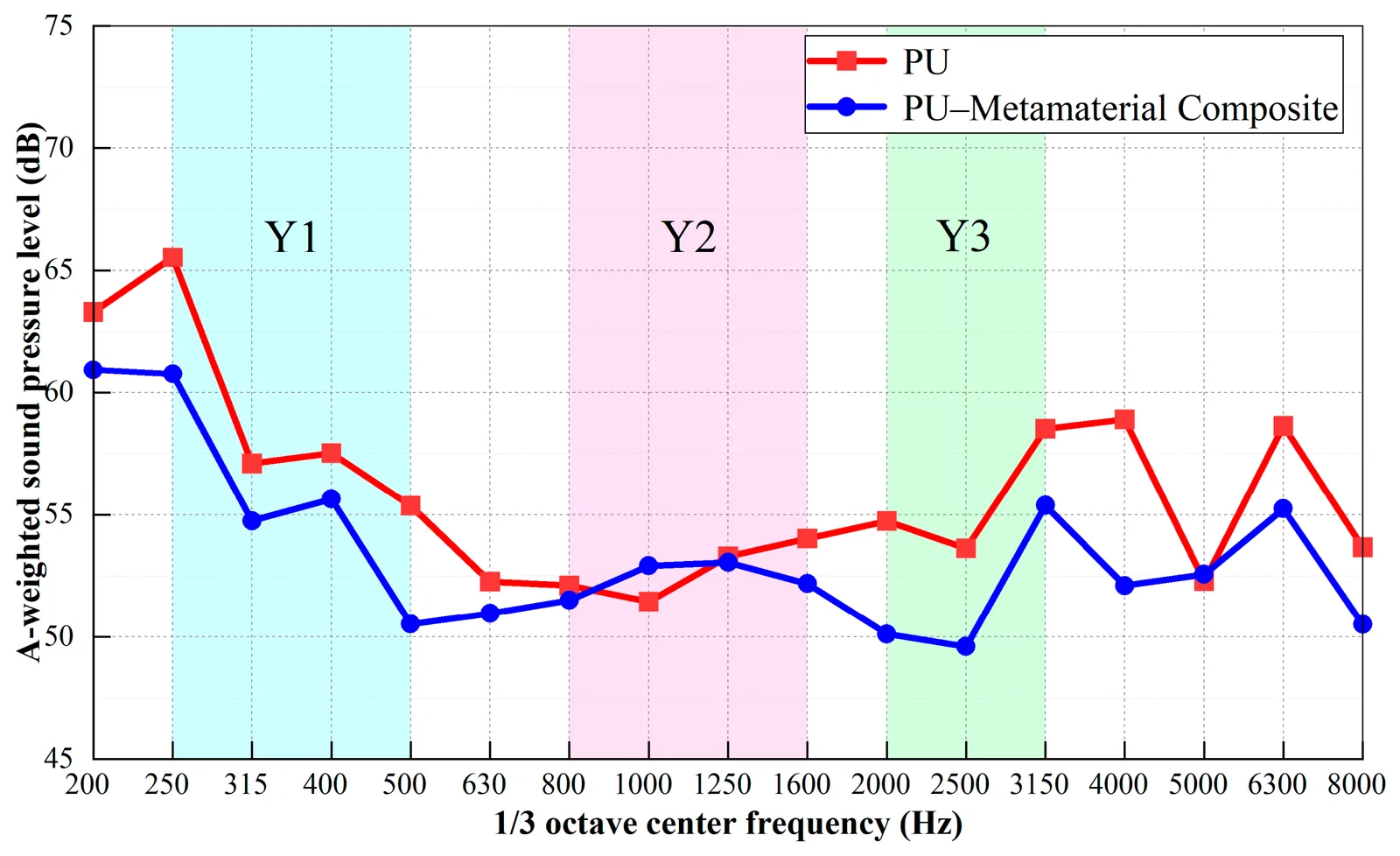
Comparison of A-weighted SPL spectra in the cab with and without the metamaterial.

After the material-level experimental validation, the measured absorption coefficient of the composite acoustic package was imported into VA One. Using the User Define Noise Control Treatment module, it was defined as a new acoustic-package material property and then applied to the target regions of the commercial vehicle cab FE–SEA model. Compared with using theoretical or idealized simulation parameters directly, a material model based on measured absorption coefficients can more realistically reflect the absorption behavior of the composite acoustic package in engineering conditions, improving the credibility of system-level noise prediction. Figure 21 shows the A-weighted SPL spectrum at the driver position before and after applying the composite acoustic package.

Overall, after the composite acoustic package was applied, the originally prominent low-frequency noise peaks inside the cab were clearly reduced. The reduction was especially significant in the low-to-mid-frequency range corresponding to the target tuning bands of the Helmholtz metamaterial, demonstrating that the metamaterial units effectively dissipate low-frequency acoustic energy along the full-vehicle noise transmission paths. Meanwhile, the PU porous layer maintained favorable broadband absorption in the mid- and high-frequency ranges, resulting in an overall downward trend across a wide frequency band. This indicates that the composite structure avoids the typical limitation of single narrowband resonant absorbers, where only isolated peaks are reduced while the inter-peak bands remain insufficiently improved.

It is worth emphasizing that the SPL reductions observed in the high-frequency bands do not indicate that the six-cavity Helmholtz structure continues to operate through its target resonance modes at high frequencies. For the 200–630 Hz low- and low-to-mid-frequency ranges, the additional attenuation is mainly associated with local resonance and thermo-viscous dissipation in the tuned sub-cavities. In the mid- and high-frequency ranges, the dominant absorption mechanism remains the viscous friction and thermal exchange within the PU porous layer. Meanwhile, the openings, partitions, and cavities of the front metamaterial layer modify the equivalent acoustic boundary, introduce local scattering and reflection, and may extend the interaction path between the incident sound field and the rear PU porous layer. Consequently, the high-frequency improvement should be interpreted as the combined effect of broadband porous dissipation in the PU layer and impedance modulation by the front metamaterial layer, rather than the high-frequency target resonance of the Helmholtz cavities.

The total A-weighted SPL provides a more direct evaluation of the noise reduction effect. The simulation results show that the total A-weighted SPL at the driver position is 81.1059 dB under the BIW condition. After applying the PU porous acoustic package, it decreases to 68.25 dB. When the Helmholtz acoustic metamaterial is further introduced, the SPL decreases to 66.01 dB. This means that the PU porous acoustic package reduces the total SPL by 12.86 dB relative to the BIW condition, and the addition of the metamaterial provides an extra reduction of 2.24 dB. Relative to the BIW condition, the final composite acoustic package achieves an overall reduction of 15.10 dB. Since the A-weighted total SPL better reflects subjective human auditory perception, these results indicate that the proposed composite acoustic package is beneficial not only at the material level and for local spectral peak control, but also for full-vehicle NVH comfort.

It should also be noted that the noise reduction gain in the system-level simulation does not exactly correspond to the improvement in impedance-tube absorption coefficient. The cabin noise response is affected not only by material absorption but also by structural transmission paths, acoustic-cavity modal distribution, local treatment regions, coverage area, and multi-source coupled excitation. Therefore, the performance of the composite acoustic package should be evaluated by combining material absorption characteristics with full-vehicle SPL responses. The present results show consistent performance at both the material-test and system-simulation levels, validating the rationality and effectiveness of the proposed design.

### 4.4. Vehicle Test and Result Analysis

To verify the practical noise reduction effect of the proposed composite acoustic package in a commercial vehicle cab, vehicle noise tests were conducted. The interior noise response at the driver position was used as the evaluation metric. Under conditions in which the vehicle operating state, test environment, and acquisition settings were kept as consistent as possible, the noise near the driver’s ear was compared between the untreated condition and the condition with the composite acoustic package installed.

Due to the limitations of the available experimental conditions, the in-vehicle test in this study was conducted as a preliminary stationary idling test. During the test, the commercial vehicle remained stationary, and the engine speed was maintained at approximately 600 rpm. The same vehicle state, microphone position, acquisition system, and analysis procedure were used for both the untreated condition and the condition with the composite acoustic package installed. Therefore, the test was mainly used to provide an engineering-level comparison of the noise reduction effect under a controlled idling condition, rather than a complete evaluation under different driving speeds and road-load excitations.

**Figure 22:**
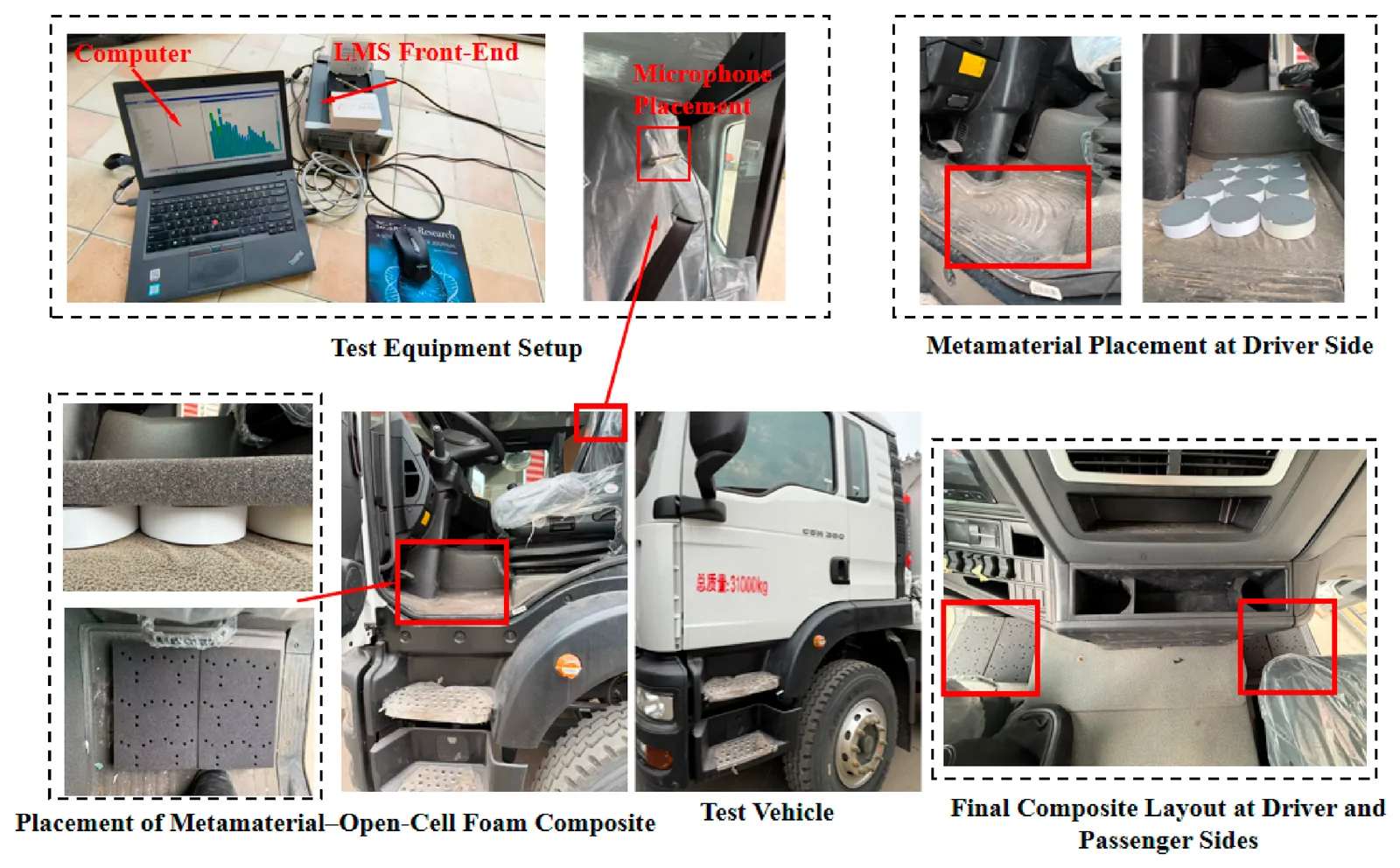
Vehicle test layout and installation of the composite acoustic package.

During the test, the microphone was placed near the driver’s ear position to approximate the driver’s actual auditory perception. This measurement point is commonly used in full-vehicle NVH evaluation and can effectively represent the subjective noise experience of the driver. The composite acoustic package was installed in the cab floor-mat region, as shown in Figure 22. This region was selected because it is close to the cab floor and constitutes an important transmission path for vibration and noise from the floor structure, road excitation, and powertrain. The preceding simulations also indicated that acoustic treatment in this area has a direct influence on the noise response at the driver position.

**Figure 23:**
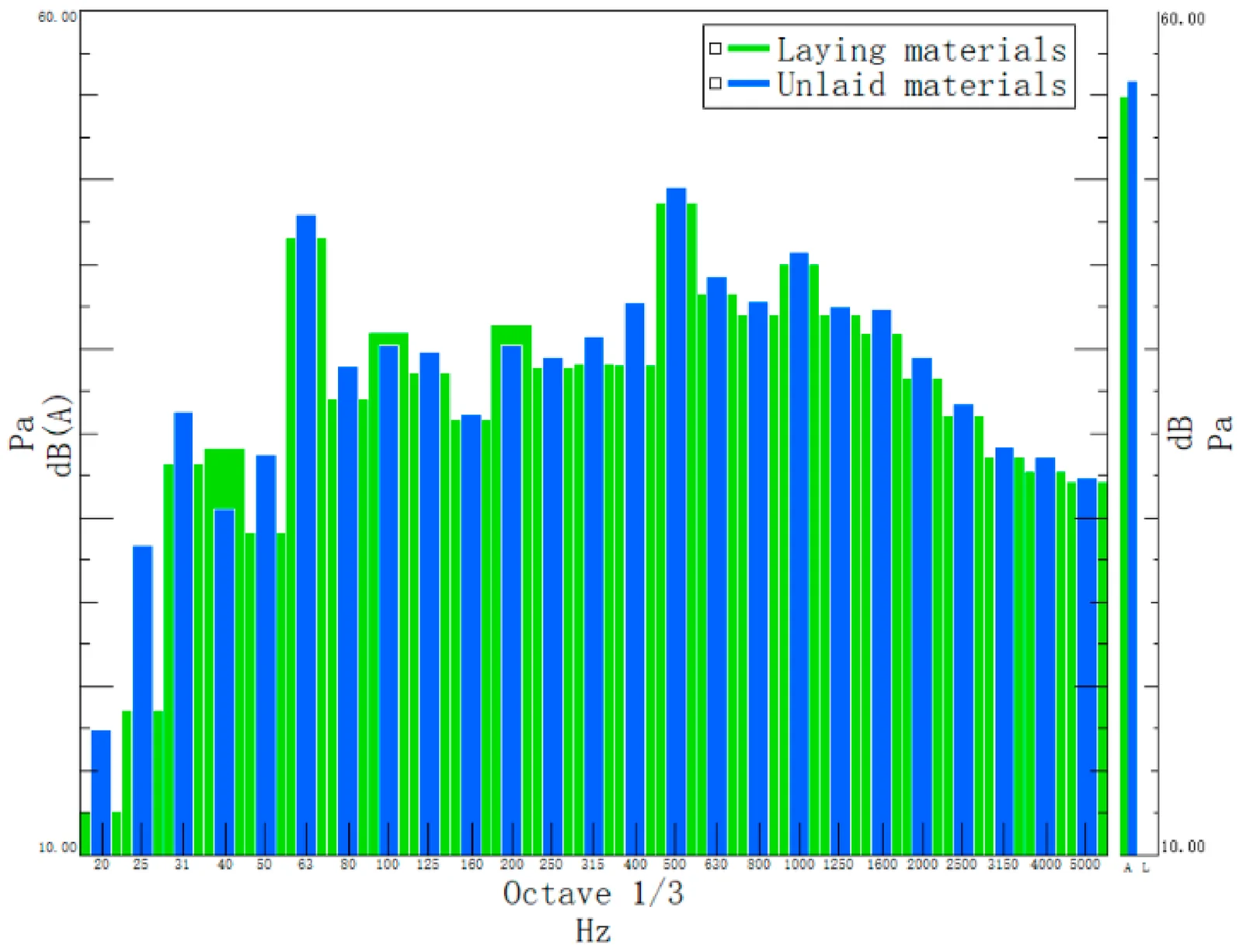
Comparison of driver-ear noise between the untreated condition and the condition with the acoustic package installed.

Based on the test arrangement, noise signals near the driver’s ear were collected for both the untreated condition and the condition with the acoustic package installed. The results were analyzed in the frequency domain and compared in terms of total SPL. Figure 23 compares the A-weighted SPL spectra near the driver’s ear under the original condition and after installing the composite acoustic package. After installation, the A-weighted noise near the driver’s ear decreases over the overall frequency range, indicating that the composite structure provides broadband noise reduction under real vehicle conditions. Multiple frequency bands show different degrees of attenuation, and some peak bands are reduced, confirming the beneficial effect of the composite acoustic package on the cab interior noise.

In terms of the total A-weighted SPL, the value near the driver’s ear decreases from 55.83 dB(A) without the acoustic package to 54.56 dB(A) after installing the composite acoustic package, corresponding to a reduction of 1.27 dB(A) under the stationary idling condition. This measured reduction is smaller than the additional reduction of 2.24 dB predicted by the VA One simulation. The difference can be attributed to several factors. First, the VA One simulation represents an equivalent acoustic-treatment condition in which the measured absorption coefficient of the composite specimen is introduced into the full-vehicle model. In this process, the local coupling between the composite acoustic package and the cab floor, edge leakage, non-uniform installation contact, and compression deformation of the PU porous layer are simplified. In the real vehicle, the contact state among the six-cavity Helmholtz metamaterial, the PU porous layer, and the floor surface is difficult to keep perfectly uniform, which may weaken the effective acoustic impedance matching and reduce the actual attenuation.

Second, the resonant response of the Helmholtz metamaterial is sensitive to the neck diameter, effective neck length, cavity volume, surface roughness, and sealing condition. Small dimensional deviations caused by 3D printing, post-processing, and assembly may shift the resonant frequencies and reduce the absorption peak amplitude compared with the ideal numerical model. Third, although the vehicle was kept under a stationary idling condition at approximately 600 rpm, the measured driver-ear noise was still affected by engine excitation, structural transmission through the floor and panels, interior reflections, background noise, and slight fluctuations in the operating state. In addition, the composite acoustic package was mainly installed in the floor-mat region, resulting in a limited treatment area. Therefore, the measured reduction of 1.27 dB(A) is more conservative than the simulated additional reduction of 2.24 dB, but it still demonstrates that the proposed composite acoustic package can produce a measurable noise-reduction effect under a real idling vehicle condition.

## 5. Conclusions

This study focuses on broadband noise control in a commercial vehicle cab and addresses the insufficient low-frequency absorption of conventional PU porous acoustic packages and the narrowband nature of single-cavity Helmholtz resonators. A PU–six-cavity Helmholtz metamaterial composite acoustic package is proposed. The study was conducted following the route of baseline noise-response identification, analysis of the basic noise-reduction effect and low-frequency limitation of the PU porous layer, parametric simulation of an embedded-neck single-cavity Helmholtz resonator, design of a six-cavity inner–outer sector acoustic metamaterial, and material-level and vehicle-level validation. The main conclusions are as follows.

(1) The baseline A-weighted sound pressure level (SPL) spectrum at the driver position was analyzed to clarify the basis for introducing the PU porous layer as the basic acoustic package. Under the body-in-white condition, the total A-weighted SPL at the driver acoustic cavity was 81.1059 dB, with pronounced noise peaks at 250 Hz, 500 Hz, and 1000 Hz. This indicates that the cab noise exhibits both broadband distribution and distinct low-frequency peaks. Considering the requirements for acoustic package installation, the PU porous layer was selected as the basic broadband sound-absorbing material to improve the mid- and high-frequency noise response and to provide a reference for the subsequent design of the low-frequency enhancement structure.
(2) The PU porous layer can provide effective broadband dissipation for mid- and high-frequency noise in the commercial vehicle cab, but its low-frequency noise-control capability remains limited. Because low-frequency sound waves have long wavelengths and the thickness of vehicle acoustic packages is constrained by the available installation space, a single PU porous layer cannot generate sufficient pore-scale velocity gradients and thermoviscous dissipation in the low-frequency range. In addition, low-frequency noise is closely related to structural panel vibration and acoustic-cavity coupling. Therefore, relying only on the PU porous material is insufficient to suppress the low- and mid-frequency peaks within 200–630 Hz, and a low-frequency enhancement structure with local resonance capability is required.
(3) A COMSOL model of an embedded-neck single-cavity Helmholtz resonator was established, and parametric simulations were performed to clarify the effects of key geometrical parameters on the absorption performance. The results show that the neck radius mainly affects the absorption peak position and peak amplitude by changing the neck cross-sectional area and the equivalent acoustic impedance matching condition. The cavity height shifts the resonant peak toward lower frequencies by changing the backing-cavity volume. The neck depth reduces the resonant frequency by increasing the effective neck length and may also broaden the absorption peak. These findings provide design guidance for the parameter allocation of the outer low-frequency cavities and inner mid-frequency cavities in the subsequent six-cavity configuration.
(4) Based on the single-cavity tuning rules, a six-cavity inner–outer sector Helmholtz acoustic metamaterial with a parallel configuration was proposed. Within a limited outer diameter of 100 mm and total thickness of 30 mm, the structure divides the unit into three outer low-frequency cavities and three inner mid-frequency cavities, corresponding to the target frequencies of 200, 250, 315, 400, 500, and 630 Hz, respectively. The three-dimensional COMSOL impedance-tube simulation results show that the six-cavity structure can form multiple absorption peaks within the target frequency range. The particle velocity distributions further indicate that, at different frequencies, the high-velocity regions are mainly concentrated around the neck and neck–cavity junction of the corresponding sub-cavity, validating the structural division of “outer-ring low-frequency cavities and inner-ring mid-frequency cavities” and the rationality of the multi-peak absorption design.
(5) A PU porous layer–six-cavity Helmholtz metamaterial composite acoustic package was constructed and validated through impedance-tube simulation, impedance-tube measurement, VA One vehicle-level simulation, and in-vehicle testing. The material-level results show that, compared with the PU-only structure, the composite structure exhibits an earlier increase in absorption and more pronounced local absorption enhancement in the low-frequency range, while maintaining the broadband dissipation capability of the PU porous layer in the mid- and high-frequency ranges. The vehicle-level simulation results show that the total A-weighted SPL at the driver position decreases from 68.25 dB for the PU porous acoustic package to 66.01 dB for the PU–six-cavity composite acoustic package, corresponding to an additional reduction of 2.24 dB. The preliminary in-vehicle test under a stationary idling condition shows that, after installing the composite acoustic package, the total A-weighted SPL near the driver’s ear decreases from 55.83 dB(A) to 54.56 dB(A), with a reduction of 1.27 dB(A). This result provides initial engineering evidence for the practical noise-reduction potential of the proposed structure, although further repeated tests under different operating conditions are still required.

Future studies will focus on diffuse-field validation, optimization of installation regions, integration with cab HVAC noise-transmission paths, and long-term service-stability evaluation, while considering structural lightweighting and manufacturing tolerance control to further improve the engineering applicability of the proposed composite acoustic package in vehicle NVH applications.

## Figures and Tables

**Table 1:** Acoustic parameters of the PU foam.

Material	Thickness (mm)	Density (kg/m^3^)	Airflow Resistivity (N·s·m^−4^)	Porosity	Tortuosity	Viscous Characteristic Length (m)	Thermal Characteristic Length (m)	Damping Loss Factor
PU	30	32	15,000	0.98	1.2	1.5 × 10^−4^	3.0 × 10^−4^	0.1

**Table 2:** COMSOL parameter settings.

Parameter	Air Density	Dynamic Viscosity	Specific Heat Ratio	Ambient Pressure	Specific Heat Capacity	Thermal Conductivity
Value	1.18	1.85 × 10^−5^	1.40	101,300	1.0 × 10^3^	2.6 × 10^−2^

**Table 3:** Parameters of the 200 Hz Helmholtz resonator.

Parameter	Value (mm)
Impedance tube radius $R_{1}$	50
Impedance tube length $L_{3}$	520
Spacing between two measurement points $L_{2}$	50
Neck radius of the embedded tube $r_{2}$	2
Embedded depth (neck length) l	17.45
Cavity height T	36
Cavity radius $r_{1}$	20
Cavity wall thickness $t_{1}$	2

**Table 4:** Neck lengths of the three outer-ring cavities.

Target Frequency	200 Hz	250 Hz	315 Hz
Effective length (mm)	24.0646	15.4013	9.07010
Physical neck length (mm)	20.6	12.0	6.3

**Table 5:** Fixed geometric parameters of the six-cavity metamaterial.

Parameter	Value
Outer diameter $D_{out}$	100 mm
Total thickness H	30 mm
Thickness of top/bottom/outer/partition walls t	2 mm
Net backing-cavity depth T	26 mm
Number of sector divisions n	3
Radial partition radius $r_{1}$	30 mm

**Table 6:** Main parameters of the six-cavity inner–outer ring structure.

Cavity Target Frequency (Hz)	Aperture Diameter d_i_ (mm)	Neck Length l_i_ (mm)
200	4	20.6
250	4	12.0
315	4	6.3
400	2.37	2
500	3.15	2
630	4.26	2

## Data Availability

The original contributions presented in this study are included in the article. Further inquiries can be directed to the corresponding authors.
